# Intranasal DC-targeting vaccine booster elicits durable and cross-clade protective immunity against sarbecoviruses in mice

**DOI:** 10.1172/JCI195784

**Published:** 2026-01-15

**Authors:** Nicholas You Zhi Cheang, Wee Chee Yap, Kirsteen McInnes Tullett, Xinlei Qian, Peck Szee Tan, Kiren Purushotorman, Wan Yi Tan, Shirley Yun Yan Mah, Paul Anthony Macary, Chee Wah Tan, Mireille Hanna Lahoud, Sylvie Alonso

**Affiliations:** 1 Infectious Diseases Translational Research Programme, Department of Microbiology and Immunology, Yong Loo Lin School of Medicine and; 2 Immunology Programme, Life Sciences Institute, National University of Singapore, Singapore.; 3 Monash Biomedicine Discovery Institute, Department of Biochemistry and Molecular Biology, Monash University, Clayton, Victoria, Australia.; 4 Immunology Translational Research Programme, Department of Microbiology and Immunology, Yong Loo Lin School of Medicine, National University of Singapore, Singapore.; 5 Communicable Diseases Agency, Singapore.

**Keywords:** Immunology, Infectious disease, Adaptive immunity, COVID-19, Vaccines

## Abstract

Short-lived, clade-specific immune responses with limited mucosal priming are limitations of current COVID-19 mRNA vaccines. We have developed a nasal booster vaccine candidate that induced robust, sustained, cross-clade, systemic, and mucosal protective immunity. Two recombinant Clec9A-specific monoclonal antibodies fused to the receptor binding domain (RBD) from Omicron XBB.1.5 and SARS-CoV-1 were generated. In Comirnaty mRNA–vaccinated mice, boosting with both constructs combined (Clec9A^OMNI^) induced cross-clade neutralizing antibodies and T cell responses that were greater in magnitude and more sustained compared with bivalent Comirnaty (BC) mRNA vaccine booster. Persistence of RBD-specific follicular helper CD4^+^ T cells, germinal center B cells, and long-lived plasma cells that facilitated affinity maturation correlated with detection of triple cross-reactive B cells binding the RBDs of ancestral SARS-CoV-2, XBB.1.5, and SARS-CoV-1. Remarkably, intranasal boosting with Clec9A^OMNI^ elicited robust and durable immunity across the upper and lower airways while concurrently boosting the systemic immunity to levels matching or exceeding those from systemic boosting. Correspondingly, Clec9A^OMNI^ nasal booster conferred superior protection against SARS-CoV-2 challenge compared with BC mRNA booster, with undetectable viral titers in the respiratory tract. Hence, Clec9A^OMNI^ is a promising nasal booster vaccine candidate that has the potential to mitigate pandemic threats from emerging sarbecoviruses.

## Abstract 2

We developed a nasal vaccine booster that induces robust and sustained, cross-clade systemic and mucosal protective immunity in COVID19mRNA vaccinated populations.

## Graphical abstract

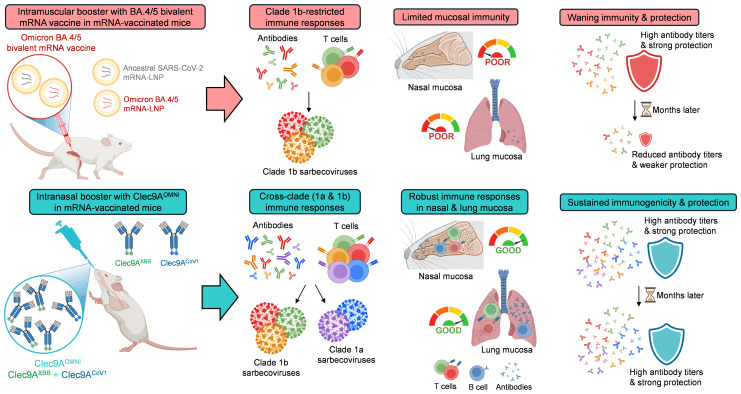

## Introduction

Sarbecoviruses are a subgenus of *Betacoronavirus* and classified into 4 distinct evolutionary clades based on their receptor binding domain (RBD) sequence in spike protein: clade 1a (SARS-CoV-1, previously referred to as SARS-CoV, and related bat sarbecoviruses), clade 1b (SARS-CoV-2, related bat and pangolin sarbecoviruses), and clades 2 and 3 (bat sarbecoviruses) (1, 2). Of concern, clade 1 members have been deemed of high zoonotic potential due to their ability to utilize human angiotensin-converting enzyme 2 (ACE2) as an entry receptor (2). Hence, clade 1a and 1b sarbecoviruses pose a tangible pandemic threat.

The majority of the world population has developed varying levels of SARS-CoV-2 immunity through repeated infections and/or vaccinations. In particular, mRNA-based COVID-19 vaccines have been administered worldwide and induce a strong neutralizing antibody (nAb) response, which has been used as a correlate of protection against symptomatic COVID-19 (3, 4). However, substantial waning of these antibodies has been observed, which rapidly reduces vaccine protective efficacy against reinfection (5). Additionally, given their i.m. route of administration, mRNA vaccines induce inadequate respiratory mucosal immunity, which is critical for protection against breakthrough infection and transmission (6, 7). The highly inflammatory nature of lipid nanoparticles that encapsulate the mRNA molecule, coupled with physical and chemical barriers within the respiratory tract, make current mRNA vaccine formulations unsuitable for i.n. delivery (8, 9). Lastly, the breadth of immune responses elicited by current mRNA COVID-19 vaccines, especially nAb, is limited toward clade 1b sarbecoviruses (10). Therefore, new-generation vaccines that can confer broad (cross-clade 1a and 1b), durable, systemic, and mucosal protective immunity are imperative to prepare against future pandemics.

In recent years, DC-targeting strategies have been increasingly explored to develop vaccine candidates against infectious diseases, including SARS-CoV-2 and sarbecoviruses (11–15). Among which, targeting vaccine antigens to the C-type lectin-like receptor Clec9A expressed on conventional type 1 DCs (cDC1 in mouse; CD141^+^ DC in humans) has demonstrated great promise in preclinical animal models by inducing potent and durable immune responses upon a single-shot immunization, which have been associated with the generation of persistent antigen-specific T follicular helper (Tfh) cell, germinal center (GC), and antibody secreting cells (ASCs) (15–20). Vaccine antigen candidates are genetically fused to the C-terminal end of each heavy chain of an anti-Clec9A mAb. The superiority of Clec9A-targeting strategy compared with other DC-targeting approaches could be partly attributed to the restricted expression of Clec9A on the cDC1 subset, thereby resulting in longer circulation time of the antibody constructs and prolonged antigen presentation (17, 18).

Previously, we engineered a Clec9A-RBD construct by fusing ancestral SARS-CoV-2 RBD to the heavy chains of anti-Clec9A mAb. We reported that single-dose Clec9A-RBD systemic immunization elicited in mice potent and sustained immune responses against all SARS-CoV-2 variants, which translated into protection upon viral challenge (15).

Here, we evaluated Clec9A-RBD immunization as a booster approach to address the limitations faced by current mRNA vaccines. We evaluated the breadth and durability of the immune responses after booster immunization in mice that received 2 doses of Pfizer-BioNTech original mRNA vaccine (Comirnaty). Two Clec9A mAb constructs were generated, containing RBD from Omicron XBB.1.5 (Clec9A^XBB^) and SARS-CoV-1 (Clec9A^CoV1^). While boosting with Clec9A^XBB^ was expected to broaden the protective immune responses to Omicron variants, boosting with Clec9A^CoV1^ was expected to provide cross-clade (1a and 1b) protective immunity, based on previous studies reporting that survivors of SARS-CoV-1 immunized with COVID-19 mRNA vaccines produced cross-clade nAb (21). Furthermore, CD103^+^ resident cDC1 expressing Clec9A is widely distributed throughout the respiratory tract, which includes the lung and nasal mucosae (22, 23). Hence, we also explored the nasal delivery route of these Clec9A-RBD constructs to induce mucosal immune responses.

## Results

### Boosting with Clec9A^XBB^ or Clec9A^CoV1^ induced durable and cross-clade immune responses, respectively.

**Figure 1:**
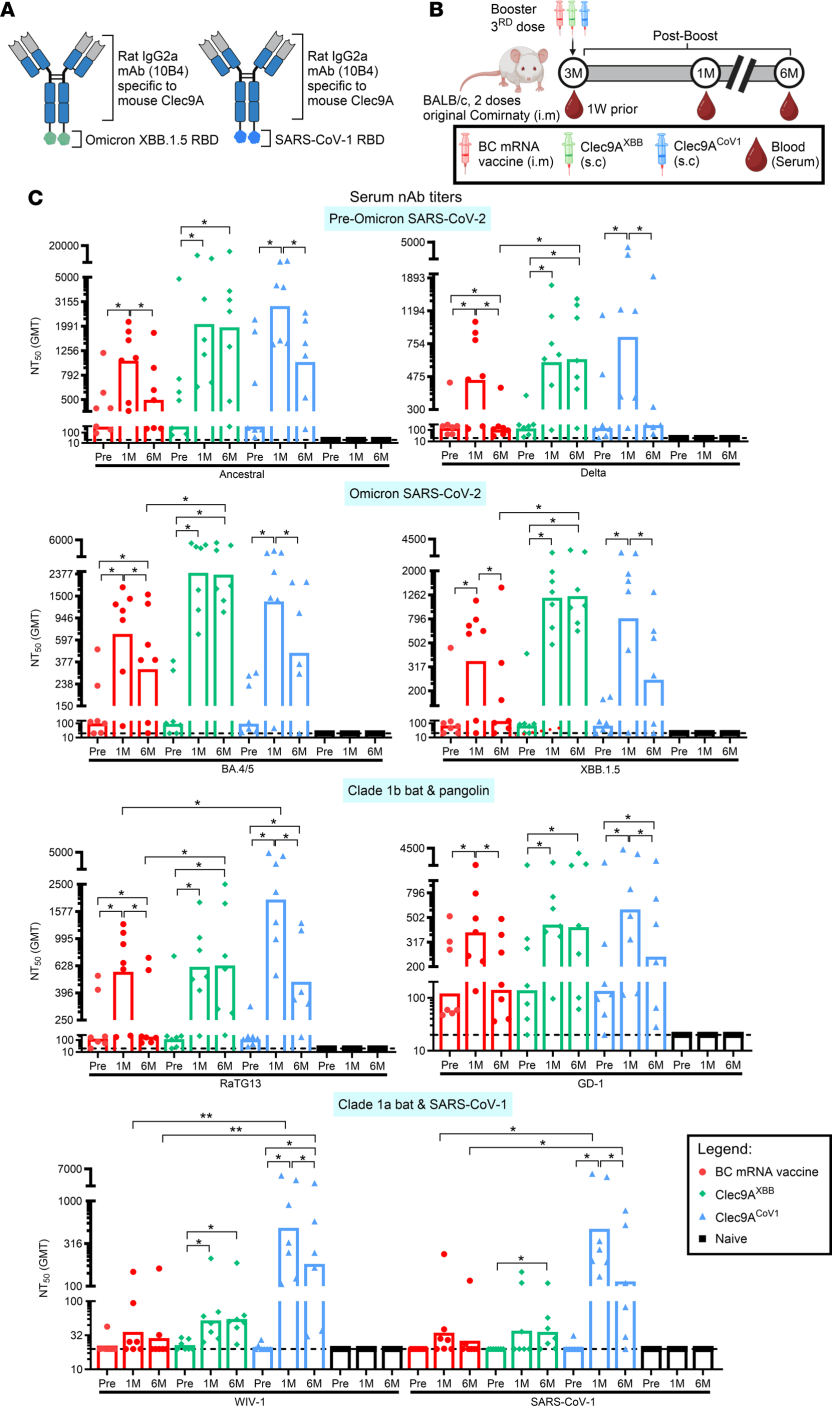
Breadth and durability of nAb responses upon systemic booster with BC mRNA vaccine, Clec9A^XBB^, and Clec9A^CoV1^. (**A**) Schematic of Clec9A^XBB^ and Clec9A^CoV1^ antibody constructs. The constructs were generated via genetic fusion of a single copy of Omicron XBB.1.5 or SARS-CoV-1 RBD antigen to each heavy chain of rat IgG2a mAb (10B4) specific to mouse Clec9A. (**B**) The 5- to 6-week-old BALB/c mice were immunized twice 3 weeks apart (0.05 μg per dose; i.m.) with Pfizer-BioNTech original Comirnaty mRNA vaccine. At 3 months after the last immunization dose, mice were boosted either with Pfizer-BioNTech BA.4/5 BC mRNA vaccine (0.05 μg; i.m.), Clec9A^XBB^ (10 μg adjuvanted with 50 μg poly I:C; s.c.), or Clec9A^CoV1^ (10 μg adjuvanted with 50 μg poly I:C; s.c.). Nonimmunized mice (naive) were also included for baseline. (**C**) Serum nAb titers against 8 sarbecoviruses from clades 1b and 1a at preboost and 1 and 6 months after boost were determined by multiplex sVNT. Data in **C** are from 1 representative experiment performed twice with similar results; *n* = 6–7 per group/experiment. Symbols represent individual animals, and data shown are geometric means. Statistical analysis: nonparametric 2-tailed Kruskal-Wallis test with Dunn’s multiple-comparison test and Friedman’s test with Dunn’s multiple-comparison test. *P < 0.05, **P < 0.01.

The breadth and durability of RBD-specific antibody responses were evaluated in mice that received 2 i.m. doses of Pfizer-BioNTech original Comirnaty mRNA vaccine 3 weeks apart, followed by systemic boosting 3 months later with Pfizer-BioNTech BA.4/5 bivalent Comirnaty (BC) mRNA vaccine (i.m.), Clec9A^XBB^ or Clec9A^CoV1^ constructs (Figure 1A); the Clec9A constructs were administered via a s.c. route and adjuvanted with polyinosinic:polycytidylic acid (poly I:C) (Figure 1B). Using a previously reported multiplex surrogate neutralizing assay (21), we observed that the 3 vaccine candidates effectively boosted the RBD-specific nAb titers (Figure 1C and Supplemental Figure 1; supplemental material available online with this article; https://doi.org/10.1172/JCI195784DS1). In addition, the nAb responses induced by Clec9A^XBB^ booster were sustained up to at least 6 months after boost, while the responses induced in BC mRNA–boosted mice waned over time, consistent with previous reports (24) (Figure 1C and Supplemental Figure 1). However, BC and Clec9A^XBB^ boosters both elicited poor cross-clade neutralization, whereby nAb activity was restricted to clade 1b sarbecoviruses (Figure 1C and Supplemental Figure 1). In contrast, boosting with Clec9A^CoV1^ generated broad systemic nAb responses against clade 1a and 1b sarbecoviruses (Figure 1C and Supplemental Figure 1). However, the nAb responses elicited by Clec9A^CoV1^ booster were not as sustained as the Clec9A^XBB^ booster (Figure 1C and Supplemental Figure 1).

To further investigate the less sustained antibody responses induced by Clec9A^CoV1^, we monitored the durability of RBD-specific immune responses upon single-shot immunization of naive mice with Clec9A^XBB^ versus Clec9A^CoV1^ (Supplemental Figure 2A). Consistent with previous studies that have reported the relative weak humoral antigenicity of Omicron XBB variant compared with other variants (25, 26), the RBD-specific IgG and nAb titers in Clec9A^XBB^-immunized mice were lower than those measured in Clec9A^CoV1^-immunized animals. However, the antibody titers persisted for a longer duration in Clec9A^XBB^-immunized mice (Supplemental Figure 2, B and C). Furthermore, the cellular immune responses triggered upon restimulation of Clec9A^CoV1^-immunized splenocytes were much stronger compared with the Clec9A^XBB^-immunized group, which were associated with robust CD4^+^ type 1 cytokine responses (Supplemental Figure 2D). Despite this strong cellular response, the RBD-specific Tfh and GC B cell responses in the spleen from Clec9A^CoV1^-immunized mice were barely detectable at 6 months after immunization, while the Clec9A^XBB^-immunized group demonstrated a much greater proportion of RBD-specific Tfh and GC B cells in their spleen at the same time point (Supplemental Figure 2, E and F). In addition, in Clec9A^CoV1^-immunized mice, RBD-specific IgG^+^ ASCs in the BM were largely skewed toward the non-long-lived plasma cell (non-LLPC) subset, in contrast with those from the Clec9A^XBB^-immunized group, which displayed increased differentiation of RBD-specific IgG^+^ ASCs into LLPCs (Supplemental Figure 2G). These observations hence provided a likely explanation for the differential persistence of the antibody responses observed between Clec9A^CoV1^ and Clec9A^XBB^-immunized mice.

Together, these results showed that Clec9A^XBB^ booster in mRNA-immunized mice elicited a sustained humoral response that was limited in breadth, while Clec9A^CoV1^ booster produced a cross-clade nAb response that did not persist as long as the Clec9A^XBB^ booster, likely due to poor intrinsic ability to induce persistent Tfh and GC B cell responses. Hence, these observations suggested that the amino acid makeup of RBD antigen influenced the immune responses.

### Systemic boosting with Clec9A^OMNI^ induced durable, cross-clade humoral responses and protected against SARS-CoV-2 challenge.

To mitigate the respective limitations faced by Clec9A^XBB^ and Clec9A^CoV1^, both constructs were combined into a single formulation, at a 1:1 or 4:1 ratio (Clec9A^XBB^/Clec9A^CoV1^) (Supplemental Figure 3A). The results indicated that both ratio formulations induced comparable nAb titers and robust cross-clade T cell responses against both sarbecovirus clades at 3 weeks after boost (Supplemental Figure 3, B and C).

**Figure 2:**
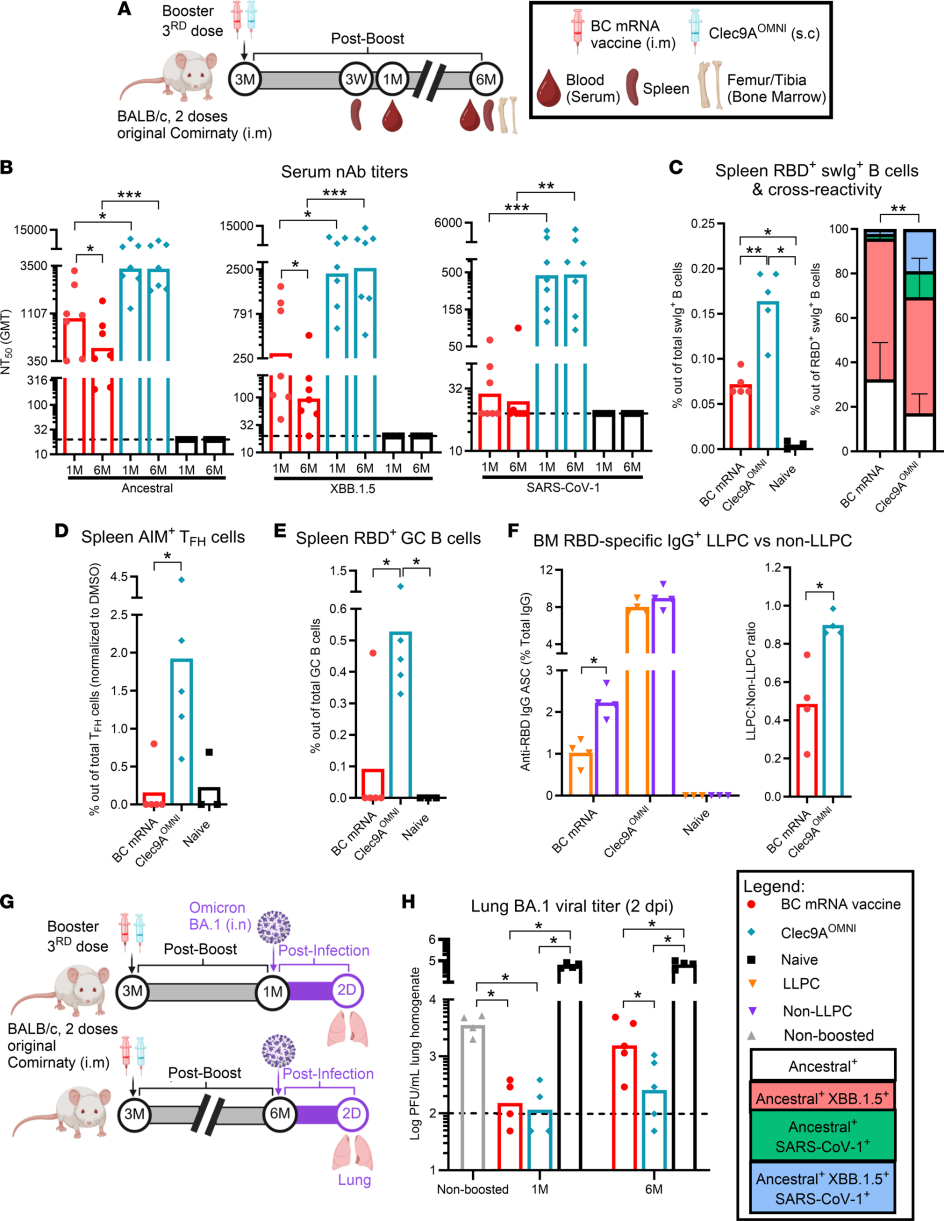
Breadth and durability of humoral responses and protection upon systemic booster with Clec9A^OMNI^ versus BC mRNA vaccine. (**A**) The 5- to 6-week-old BALB/c mice were immunized with original Comirnaty mRNA vaccine as described in Figure 1. At 3 months after the last immunization, mice were boosted with either BC mRNA vaccine (0.05 μg; i.m.) or Clec9A^OMNI^ (8 μg Clec9A^XBB^ + 2 μg Clec9A^CoV1^ adjuvanted with 50 μg poly I:C; s.c.). (**B**) Blood was collected at 1 and 6 months after boost, and serum nAb titers against ancestral SARS-CoV-2, XBB.1.5, and SARS-CoV-1 were determined by multiplex sVNT. (**C**) Percentage of ancestral SARS-CoV-2 RBD^+^ swIg^+^ B cells in the spleen was determined by flow cytometry at 3 weeks after boost. Cross-reactivity of antigen-specific B cells toward XBB.1.5 and SARS-CoV-1 RBD is also shown. (**D**–**F**) Spleen and BM from femur/tibia were harvested at 6 months after boost. (**D** and **E**) Percentages of AIM^+^ Tfh (**D**) and RBD^+^ GC (**E**) B cells in spleen were determined by flow cytometry. (**F**) Frequency of BM RBD-specific IgG^+^ LLPCs and non-LLPCs (normalized to total IgG) was determined by B cell ELISpot. (**G**) At 1 and 6 months after boost, mice were challenged with Omicron BA.1 (10^6^ PFU; i.n.). At 2 dpi, lungs were harvested and homogenized. (**H**) BA.1 lung homogenate viral titers were quantified via plaque assay. Data in **B**–**F** and **H** are from 1 representative experiment performed twice with similar results; *n* = 4–7 per group/experiment. Symbols represent individual animals, and data shown are geometric means (**B**) and means (**C**–**F** and **H**) ± SD (**C**). Statistical analysis: nonparametric 2-tailed Mann-Whitney test (**B**, **C**, and **F**), Wilcoxon’s matched-pairs signed rank test (**B**), and Kruskal-Wallis test with Dunn’s multiple-comparison test (**C**–**E** and **H**). *P < 0.05, **P < 0.01, ***P < 0.001.

Using the 4:1 dose combination (hereby referred to as Clec9A^OMNI^), we carried out in-depth characterization of the breadth and durability of the humoral immune responses induced upon s.c. boosting (Figure 2A). Results indicated that boosting with Clec9A^OMNI^ induced cross-clade systemic nAb responses that were sustained for at least 6 months after boost compared with immune sera from BC mRNA–boosted mice, which displayed limited neutralizing activity against clade 1a sarbecoviruses and waning of nAb titers over time (Figure 2B and Supplemental Figure 4). Additionally, a unique subset of RBD-specific switched Ig^+^ (swIg^+^) B cells that were cross-reactive toward ancestral SARS-CoV-2, XBB.1.5, and SARS-CoV-1 RBD were detected in the spleen from Clec9A^OMNI^-boosted mice (Figure 2C). In contrast, the cross-reactivity of RBD-specific swIg^+^ B cells in the spleen from mice boosted with BC mRNA vaccine was restricted to ancestral SARS-CoV-2 and XBB.1.5 RBD only (Figure 2C). Moreover, the durable nAb response elicited by Clec9A^OMNI^ booster correlated with the greater proportion of RBD-specific Tfh and GC B cells in the spleen (Figure 2, D and E), coupled with greater frequencies of antigen-specific LLPCs in the BM at 6 months after boost compared with boosting with BC mRNA vaccine, which were barely detectable and more skewed toward the non-LLPC subset (Figure 2F).

The protective efficacy of Clec9A^OMNI^ systemic booster was also investigated. Mice were challenged nasally with Omicron BA.1 virus at either 1 or 6 months after boost, and the lung viral titers were measured (Figure 2G). Compared with nonboosted animals, mice boosted with either BC or Clec9A^OMNI^ had comparable and substantially reduced lung viral titers upon challenge performed at 1 month after boost (Figure 2H). However, when the challenge was performed at 6 months after boost, the lung viral titers were much lower in Clec9A^OMNI^-boosted mice compared with BC mRNA–boosted mice (Figure 2H).

Together, these data indicated that s.c. booster immunization with Clec9A^OMNI^ induced cross-clade and durable nAb responses that conferred superior long-term protection than BC mRNA booster.

### Nasal boosting with Clec9A^OMNI^ generated robust, cross-clade humoral and cellular responses in both systemic and mucosal compartments.

**Figure 3:**
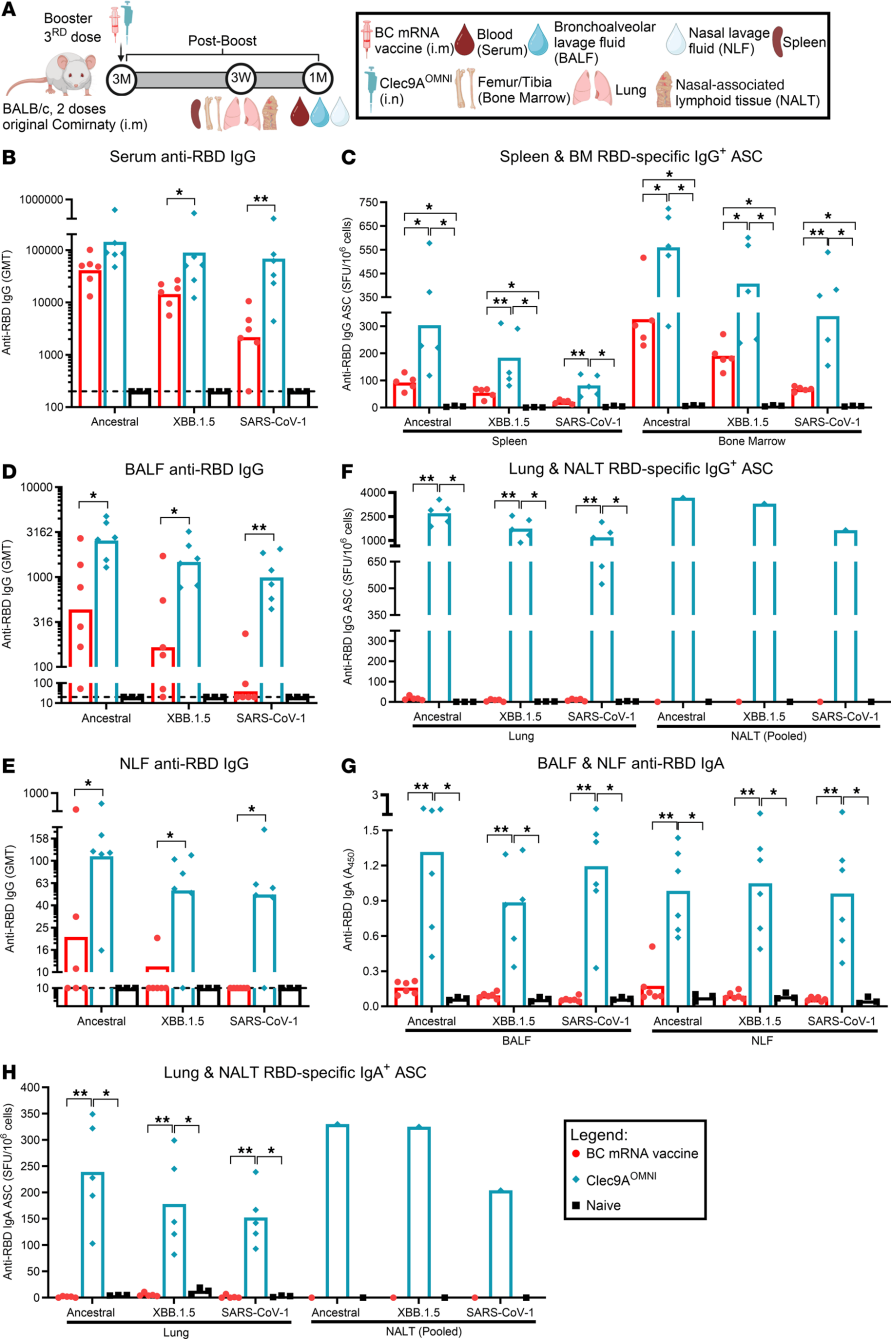
IgG and IgA responses upon nasal booster with Clec9A^OMNI^ versus systemic booster with BC mRNA vaccine. (**A**) The 5- to 6-week-old BALB/c mice were immunized with original Comirnaty mRNA vaccine as described in Figure 1. At 3 months after the second immunization, mice were boosted with either BC mRNA vaccine (0.05 μg; i.m.) or Clec9A^OMNI^ (4 μg Clec9A^XBB^ + 1 μg Clec9A^CoV1^ adjuvanted with 50 μg poly I:C; i.n.). (**B**, **D**, **E**, and **G**) At 1 month after boost, mice were euthanized to collect blood, BALF, and NLF. Serum (**B**), BALF (**D**), and NLF (**E**) anti-RBD IgG titers, and BALF and NLF (**G**) anti-RBD IgA response against ancestral SARS-CoV-2, XBB.1.5, and SARS-CoV-1 RBD were determined by ELISA. (**C**, **F**, and **H**) Spleen, BM from femur/tibia, lung, and NALT were harvested at 3 weeks after boost. Frequency of spleen and BM RBD-specific IgG^+^ ASCs (**C**), lung and pooled NALT RBD-specific IgG^+^ ASCs (**F**), and lung and pooled NALT RBD-specific IgA^+^ ASCs (**H**) reactive to ancestral SARS-CoV-2, XBB.1.5, and SARS-CoV-1 RBD was determined by B cell ELISpot. Data in **B**–**H** are from 1 representative experiment performed twice with similar results; *n* = 5–6 per group/experiment. Symbols represent individual animals, and data shown are geometric means (**B**, **D**, and **E**) and means (**C** and **F**–**H**). Statistical analysis: nonparametric 2-tailed Mann-Whitney test (**B**, **D**, and **E**) and Kruskal-Wallis test (**C** and **F**–**H**) with Dunn’s multiple-comparison test. *P < 0.05, **P < 0.01.

The ability to induce protective immunity in the respiratory mucosa represents a highly desired and notable advancement over current systemic vaccination methods for providing strong protection and reducing transmission between individuals. Thus, we investigated the suitability of the nasal route of Clec9A^OMNI^ booster immunization (Figure 3A). At 1 month after boost, BC mRNA vaccine and Clec9A^OMNI^ boosters both triggered strong serum anti-RBD IgG responses, with the latter inducing greater binding antibody titers against SARS-CoV-1 RBD (Figure 3B). Consistently, both boosters produced RBD-specific IgG^+^ ASCs in the spleen and BM, where ASCs in BC mRNA–boosted mice were mainly reactive to ancestral SARS-CoV-2 and XBB.1.5 RBD, while Clec9A^OMNI^ booster elicited higher frequency of ASCs that were reactive to ancestral SARS-CoV-2, XBB.1.5, and SARS-CoV-1 RBD (Figure 3C).

Low levels of anti-RBD IgG were detected in the bronchoalveolar lavage fluid (BALF) and nasal lavage fluid (NLF) from BC mRNA–boosted mice, while Clec9A^OMNI^ booster instead generated higher anti-RBD IgG titers against ancestral SARS-CoV-2, XBB.1.5, and SARS-CoV-1 RBD in the BALF and NLF (Figure 3, D and E). Moreover, RBD-specific IgG^+^ ASCs were undetectable in the lung and nasal-associated lymphoid tissue (NALT) from mice boosted with BC mRNA vaccine (Figure 3F), suggesting that IgG detected in the lavage fluids was spillover from the circulation, as previously proposed (7, 9, 27). Uniquely, triple cross-reactive RBD-specific IgG^+^ ASCs were detected in the lung and NALT from Clec9A^OMNI^-boosted mice (Figure 3F), supporting successful priming of antigen-specific B cells and antibody responses in the respiratory mucosa. Furthermore, while undetectable following BC mRNA vaccine booster, nasal delivery of Clec9A^OMNI^ booster induced anti-RBD IgA in BALF and NLF, coupled with RBD-specific IgA^+^ ASCs in the lung and NALT (Figure 3, G and H).

**Figure 4:**
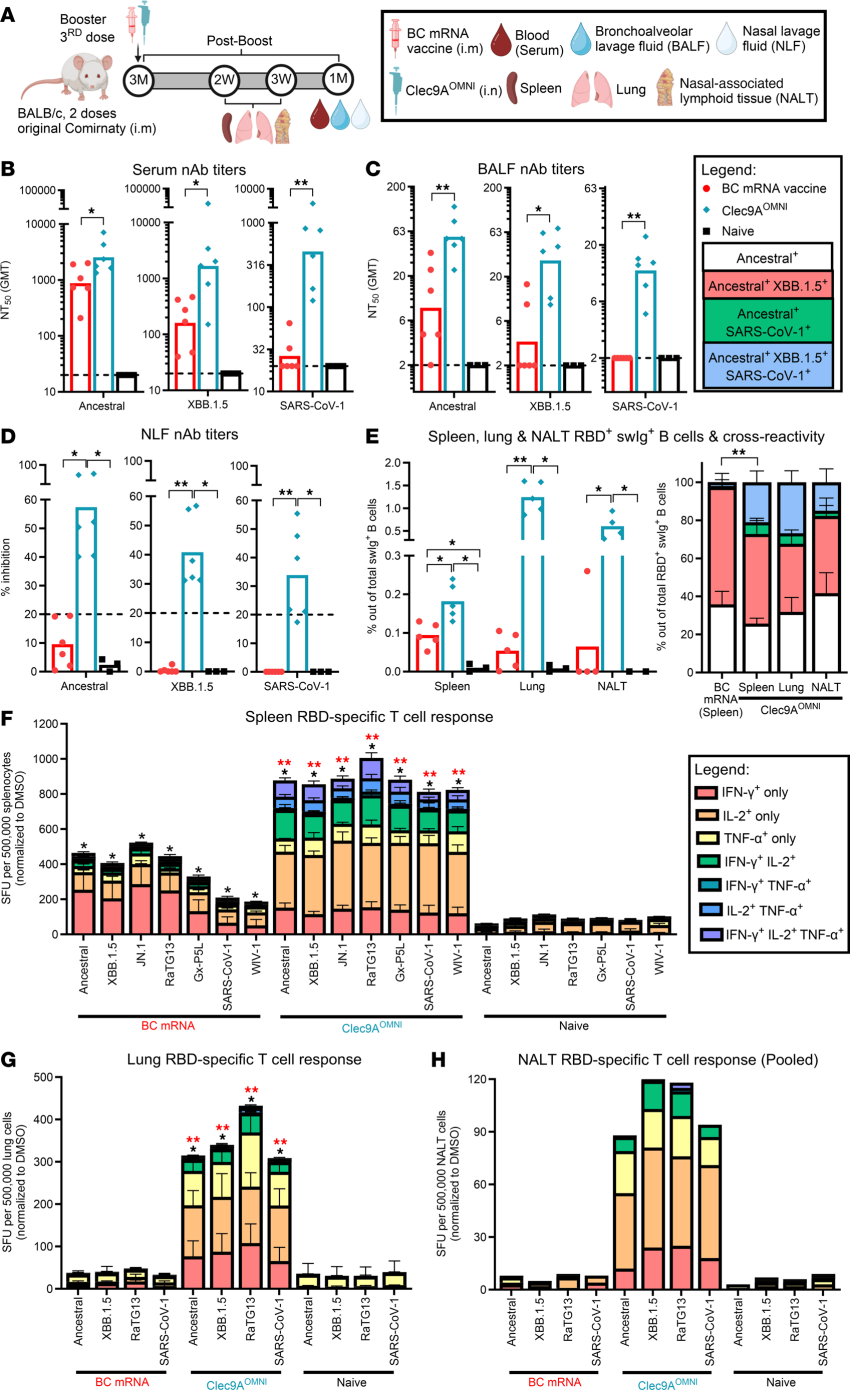
Breadth of nAb and cellular responses upon nasal booster with Clec9A^OMNI^ versus systemic booster with BC mRNA vaccine. (**A**) The 5- to six-week-old BALB/c mice were immunized with original Comirnaty mRNA vaccine as described in Figure 1. At 3 months after the second immunization dose, mice were boosted with either BC mRNA vaccine (0.05 μg; i.m.) or Clec9A^OMNI^ (4 μg Clec9A^XBB^ + 1 μg Clec9A^CoV1^ adjuvanted with 50 μg poly I:C; i.n.). (**B**–**D**) At 1 month after boost, blood, BALF, and NLF were collected. Serum (**B**), BALF (**C**), and NLF (**D**) nAb titers against ancestral SARS-CoV-2, XBB.1.5, and SARS-CoV-1 were determined by multiplex sVNT. (**E**–**H**) Spleen, lung, and NALT were harvested at 2 and 3 weeks after boost. (**E**) Percentage of ancestral SARS-CoV-2 RBD^+^ swIg^+^ B cells in spleen, lung, and NALT at 3 weeks after boost was determined by flow cytometry. Cross-reactivity of antigen-specific B cells toward XBB.1.5 and SARS-CoV-1 RBD is also shown. (**F**–**H**) Frequency of IFN-γ–, IL-2–, and/or TNF-α–secreting splenocytes (**F**), lung cells (**G**), and pooled NALT cells (**H**) at 2 weeks after boost was determined by FluoroSpot upon restimulation with ancestral SARS-CoV-2, XBB.1.5, JN.1, RaTG13, Gx-P5L, SARS-CoV-1, and WIV-1 RBD peptides. Data in **B**–**H** are from 1 representative experiment performed twice with similar results; *n* = 4–6 per group/experiment. Symbols in **B**–**E** represent individual animals, and data shown are geometric means (**B** and **C**) and means (**D**–**H**) ± SD (**E**–**G**). Statistical analysis: nonparametric 2-tailed Mann-Whitney test (**B**, **C**, and **E**) and Kruskal-Wallis test (**D** and **F**–**H**) with Dunn’s multiple-comparison test. *P < 0.05, **P < 0.01. Asterisk colors in **F** and **G** represent statistical significance between corresponding groups.

The systemic and respiratory nAb responses were also measured at 1 month after boost (Figure 4A). Although i.m. BC mRNA vaccine booster induced potent systemic nAb responses against clade 1b sarbecoviruses, very limited neutralizing activities were observed in the BALF and NLF (Figure 4, B–D, and Supplemental Figure 5). On the contrary, i.n. Clec9A^OMNI^ booster immunization elicited robust and cross-clade serum, BALF, and NLF nAb responses (Figure 4, B–D, and Supplemental Figure 5). Here again, the detection of triple cross-reactive swIg^+^ B cells binding to RBDs from ancestral SARS-CoV-2, XBB.1.5, and SARS-CoV-1 in the spleen, lung, and NALT of Clec9A^OMNI^-boosted mice may partially explain the broad nAb activity measured in these mice (Figure 4E). In contrast, BC mRNA vaccine booster displayed undetectable levels of RBD-specific swIg^+^ B cells in the lung and NALT (Figure 4E).

The antigen-specific systemic and mucosal T cell responses were also characterized 2 weeks after i.n. boosting with Clec9A^OMNI^, relative to BC i.m. booster. Although both boosters elicited detectable RBD-specific systemic cellular responses upon restimulation of splenocytes with sarbecovirus RBD peptides from both clades 1a and 1b, these responses were much stronger and more polyfunctional in Clec9A^OMNI^-boosted mice compared with BC mRNA–boosted mice (Figure 4F). Additionally, while undetectable in the BC boosted group, mice i.n. boosted with Clec9A^OMNI^ produced cross-clade RBD-specific mono- and polyfunctional T cell responses in the lung and NALT (Figure 4, G and H). Furthermore, analysis of the CD4^+^ and CD8^+^ subsets revealed that the cross-clade polyfunctional systemic and mucosal RBD-specific T cell response in Clec9A^OMNI^-boosted mice was CD4^+^ dominant, while BC mRNA–boosted mice displayed a clade 1b–restricted RBD-specific cellular response that was slightly skewed toward the CD8^+^ subset (Supplemental Figure 6).

Together, the results demonstrated that i.n. booster immunization with Clec9A^OMNI^ elicited more robust and broader RBD-specific humoral and polyfunctional T cell responses against clade 1a and 1b sarbecoviruses in both systemic and mucosal (upper and lower respiratory tract) compartments, compared with i.m. BC mRNA booster.

### Nasal boosting with Clec9A^OMNI^ was superior to systemic Clec9A^OMNI^ booster and nontargeting nasal booster.

**Figure 5:**
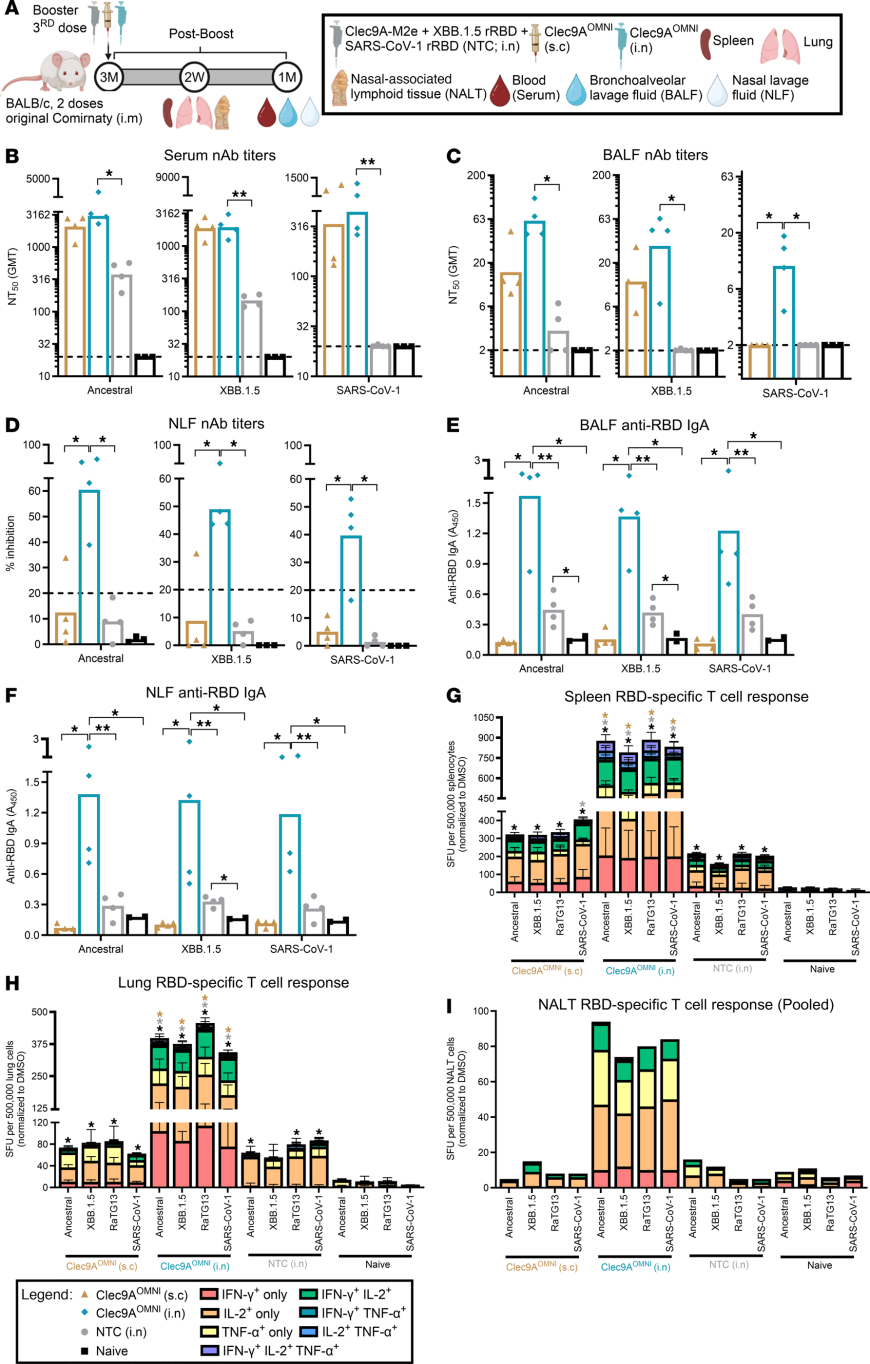
Comparison of nasal boosting with Clec9A^OMNI^ versus systemic or nontargeting boosting. (**A**) The 5- to 6-week-old BALB/c mice were immunized with original Comirnaty mRNA vaccine as described in Figure 1. At 3 months after the second immunization dose, mice were boosted with either Clec9A^OMNI^ (4 μg Clec9A^XBB^ + 1 μg Clec9A^CoV1^) (i.n. or s.c.) or with an equivalent antigen dose of purified XBB.1.5 (1 μg) and SARS-CoV-1 (0.25 μg) rRBD, in combination with Clec9A-M2e (3.75 μg) (NTC). All the formulations were adjuvanted with 50 μg poly I:C. (**B**–**F**) Blood, BALF, and NLF were obtained at 1 month after boost. Serum (**B**), BALF (**C**), and NLF (**D**) nAb titers against ancestral SARS-CoV-2, XBB.1.5, and SARS-CoV-1 were determined by multiplex sVNT. BALF (**E**) and NLF (**F**) anti-RBD IgA response against ancestral SARS-CoV-2, XBB.1.5, and SARS-CoV-1 RBD was determined by ELISA. (**G**–**I**) Spleen, lung, and NALT were harvested at 2 weeks after boost. Frequency of IFN-γ–, IL-2–, and/or TNF-α–secreting splenocytes (**G**), lung cells (**H**), and pooled NALT cells (**I**) was determined by FluoroSpot upon restimulation with ancestral SARS-CoV-2, XBB.1.5, RaTG13, and SARS-CoV-1 RBD peptides. Data in **B**–**I** are from 1 representative experiment performed twice with similar results; *n* = 4 per group/experiment. Symbols in **B**–**F** represent individual animals, and data shown are geometric means (**B** and **C**) and means (**D**–**I**) ± SD (**G** and **H**). Statistical analysis: nonparametric 2-tailed Mann-Whitney test (**B** and **C**) and Kruskal-Wallis test (**D**–**H**) with Dunn’s multiple-comparison test. *P < 0.05, **P < 0.01. Asterisk colors in **G** and **H** represent statistical significance between corresponding groups.

The immunogenicity of Clec9A^OMNI^ nasal booster was next directly compared with (a) Clec9A^OMNI^ systemic boosting and (b) nasal boosting with a nontargeting construct (NTC) (Figure 5A), the latter consisting of an equivalent antigen dose of recombinant XBB.1.5 and SARS-CoV-1 RBD (rRBD), combined with an irrelevant Clec9A-targeting construct (Clec9A-M2e), adjuvanted with poly I:C.

Upon comparing the 2 routes of Clec9A^OMNI^ boosting (s.c. versus i.n.), results indicated that both induced comparable systemic nAb titers against ancestral SARS-CoV-2, XBB.1.5, and SARS-CoV-1 (Figure 5B), while the nAb titers and anti-RBD IgA levels measured in the lungs and nasal tissues were greater in mice that were boosted via the i.n. route (Figure 5, C–F). Furthermore, the magnitude of the cellular immune responses in the spleen, lungs, and NALT was much greater in nasally boosted mice (Figure 5, G–I).

Upon comparing nasal boosting with Clec9A^OMNI^ versus NTC, systemic and mucosal nAb titers, anti-RBD IgA titers, and RBD-specific cellular immune responses were greater in Clec9A^OMNI^-boosted mice (Figure 5, B–I).

Together, these data demonstrated the superiority of the Clec9A-targeting nasal boosting approach in mounting robust antigen-specific immune responses in the upper and lower respiratory mucosa, while inducing a potent systemic recall response.

### Nasal boosting with Clec9A^OMNI^ induced durable immune responses.

**Figure 6:**
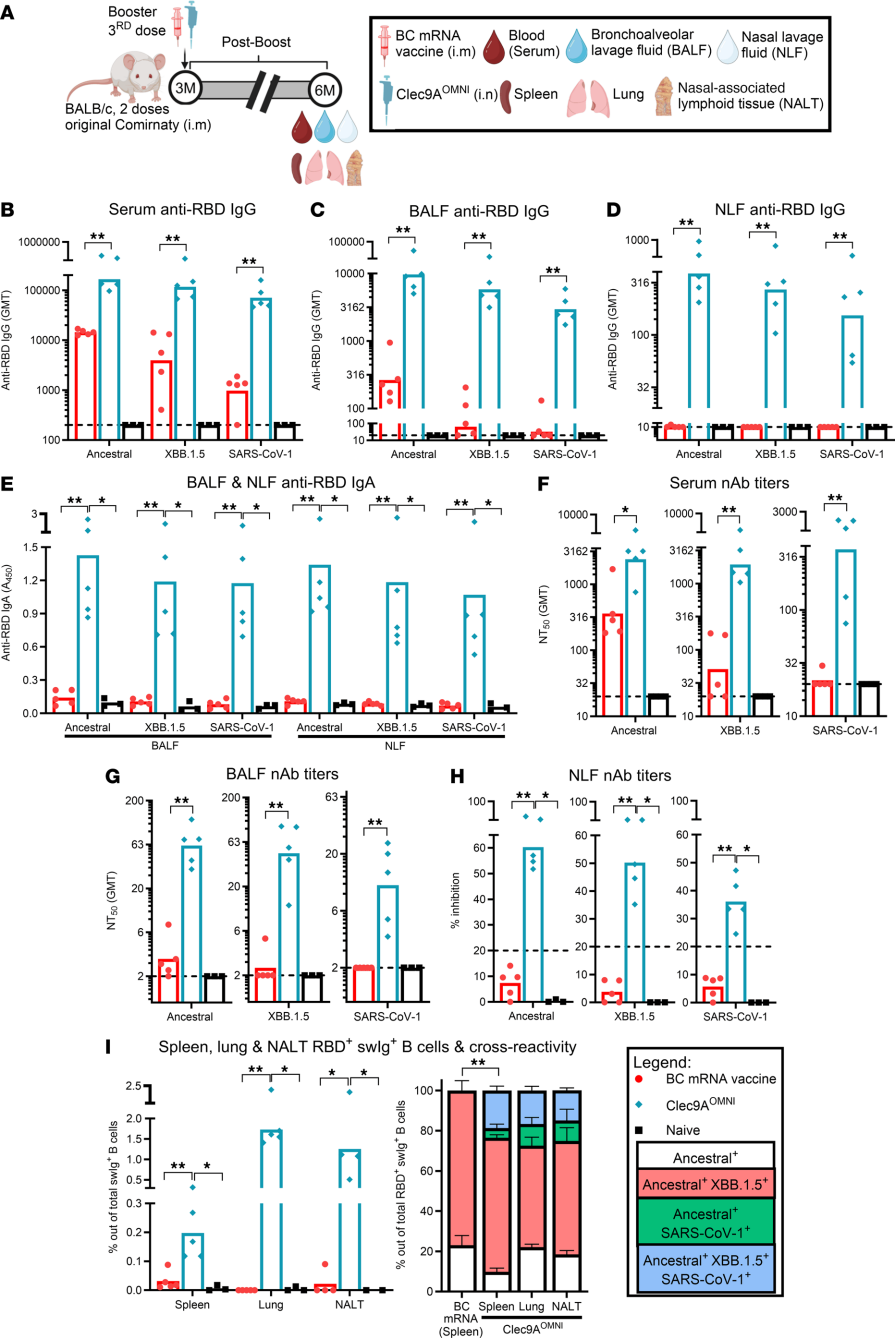
Durability of antibody responses upon nasal booster with Clec9A^OMNI^ versus systemic booster with BC mRNA vaccine. (**A**) The 5- to six-week-old BALB/c mice were immunized with original Comirnaty mRNA vaccine as described in Figure 1. At 3 months after the second immunization dose, mice were boosted with either BC mRNA vaccine (0.05 μg; i.m.) or Clec9A^OMNI^ (4 μg Clec9A^XBB^ + 1 μg Clec9A^CoV1^ adjuvanted with 50 μg poly I:C; i.n.). (**B**–**E**) Blood, BALF, NLF, spleen, lung, and NALT were obtained at 6 months after boost. Serum (**B**), BALF (**C**), and NLF (**D**) anti-RBD IgG titers, and BALF and NLF anti-RBD IgA (**E**) response against ancestral SARS-CoV-2, XBB.1.5, and SARS-CoV-1 RBD were determined by ELISA. (**F**–**H**) Serum (**F**), BALF (**G**), and NLF (**H**) nAb titers against ancestral SARS-CoV-2, XBB.1.5, and SARS-CoV-1 were determined by multiplex sVNT. (**I**) Percentage of ancestral SARS-CoV-2 RBD^+^ swIg^+^ B cells in spleen, lung, and NALT was determined by flow cytometry. Cross-reactivity of antigen-specific B cells toward XBB.1.5 and SARS-CoV-1 RBD is also shown. Data in **B**–**I** are from 1 representative experiment performed twice with similar results; *n* = 5 per group/experiment. Symbols in **B**–**I** represent individual animals, and data shown are geometric means (**B**–**D**, **F**, and **G**) and means (**E**, **H**, and **I**). Statistical analysis: nonparametric 2-tailed Mann-Whitney test (**B**–**D**, **F**, **G**, and **I**) and Kruskal-Wallis test (**E**, **H**, and **I**) with Dunn’s multiple-comparison test. *P < 0.05, **P < 0.01.

The antibody responses induced upon Clec9A^OMNI^ nasal booster were next evaluated at 6 months after boost (Figure 6A). Potent IgG responses specific to ancestral SARS-CoV-2, XBB.1.5, and SARS-CoV-1 RBD were detected in the serum, BALF, and NLF from i.n. Clec9A^OMNI^-boosted animals, with titers that were higher than those measured in i.m. BC mRNA–boosted animals (Figure 6, B–D). Of note, while the antibody titers measured in BC mRNA–boosted mice waned over time but persisted in Clec9A^OMNI^-boosted animals, the difference in titers between both boosted groups was greater at 6 months after boost than at 1 month after boost (Figure 3, B, D, and E). Moreover, while still undetectable in BC mRNA–boosted mice, cross-clade anti-RBD IgA responses persisted in the BALF and NLF from Clec9A^OMNI^-boosted mice at 6 months after boost (Figure 6E).

Similarly, robust systemic and mucosal cross-clade nAb responses were still detected in the serum, BALF, and NLF of Clec9A^OMNI^-boosted mice at 6 months after boost, while BC mRNA–boosted mice displayed much lower potency and breadth in serum nAb responses, coupled with largely undetectable neutralizing activities in the BALF and NLF (Figure 6, F–H, and Supplemental Figure 7, A–C). These observations were consistent with the persistence of triple cross-reactive RBD-specific swIg^+^ B cells toward ancestral SARS-CoV-2, XBB.1.5, and SARS-CoV-1 RBD in the spleen, lung, and NALT from Clec9A^OMNI^-boosted mice compared with the limited presence of cross-reactive RBD-specific swIg^+^ B cells in all 3 immune compartments of BC mRNA–boosted mice (Figure 6I).

**Figure 7:**
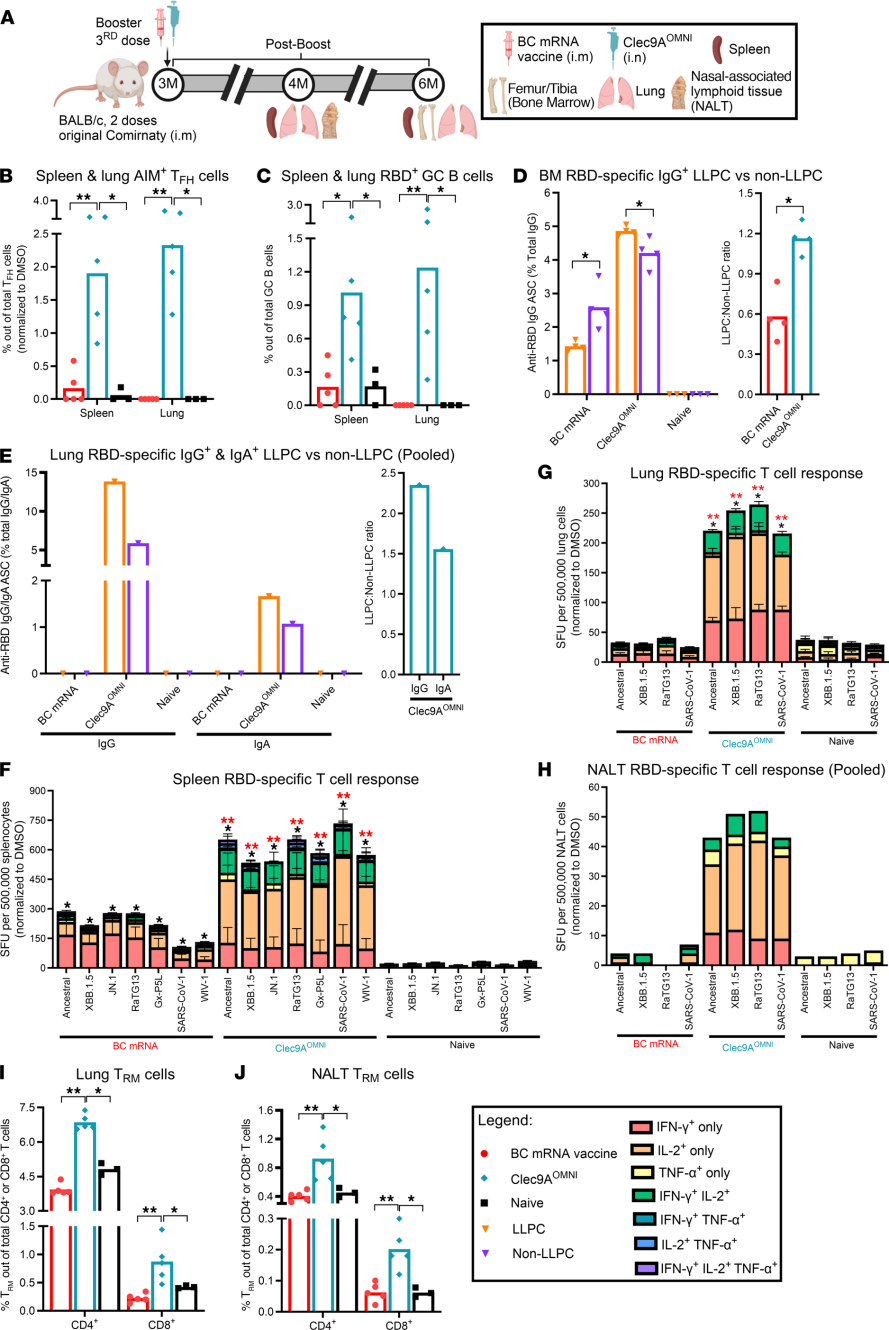
Durability of humoral and cellular responses upon nasal booster with Clec9A^OMNI^ versus systemic booster with BC mRNA vaccine. (**A**) The 5- to 6-week-old BALB/c mice were immunized with original Comirnaty mRNA vaccine as described in Figure 1. At 3 months after the second immunization dose, mice were boosted with either BC mRNA vaccine (0.05 μg; i.m.) or Clec9A^OMNI^ (4 μg Clec9A^XBB^ + 1 μg Clec9A^CoV1^ adjuvanted with 50 μg poly I:C; i.n.). (**B**–**E**) Spleen, BM from femur/tibia, and lung were harvested at 6 months after boost. (**B** and **C**) Percentages of AIM^+^ Tfh (**B**) and RBD^+^ GC (**C**) B cells in spleen and lung were determined by flow cytometry. (**D** and **E**) Frequency of BM RBD-specific IgG^+^ LLPCs and non-LLPCs (normalized to total IgG) (**D**) and pooled lung RBD-specific IgG^+^ and IgA^+^ LLPCs and non-LLPCs (normalized to total IgG and IgA) (**E**) was determined by B cell ELISpot. (**F**–**J**) Spleen, lung, and NALT were harvested at 4 months after boost. (**F**–**H**) Frequencies of IFN-γ–, IL-2–, and/or TNF-α–secreting splenocytes (**F**), lung cells (**G**), and pooled NALT cells (**H**) were determined by FluoroSpot upon restimulation with ancestral SARS-CoV-2, XBB.1.5, JN.1, RaTG13, Gx-P5L, SARS-CoV-1, and WIV-1 RBD peptides. (**I** and **J**) Percentages of lung (**I**) and NALT (**J**) CD4^+^ and CD8^+^ T_RM_ cells were determined by flow cytometry. Data in **B**–**J** are from 1 representative experiment performed twice with similar results; *n* = 4–5 per group/experiment. Symbols in **B**–**E**, **I**, and **J** represent individual animals, and data shown in **B**–**J** are means. Statistical analysis: nonparametric 2-tailed Mann-Whitney test (**D**) and Kruskal-Wallis test (**B**, **C**, **F**, **G**, **I**, and **J**) with Dunn’s multiple-comparison test. *P < 0.05, **P < 0.01. Asterisk colors in **F** and **G** represent statistical significance between corresponding groups.

We also evaluated the RBD-specific cellular responses at 4 and 6 months after boost (Figure 7A). The durable systemic and mucosal antibody responses following i.n. boosting with Clec9A^OMNI^ correlated with the presence of RBD-specific Tfh and GC B cells in the spleen and lung at 6 months after boost, while BC mRNA–boosted mice demonstrated limited presence of these cellular subsets in both compartments (Figure 7, B and C). Additionally, a greater proportion of RBD-specific BM IgG^+^ LLPCs, coupled with lung IgG^+^ and IgA^+^ LLPCs, were elicited in Clec9A^OMNI^-boosted mice compared with BC mRNA vaccine booster, which induced RBD-specific BM ASCs skewed toward the non-LLPC subset and no detectable RBD-specific lung ASCs (Figure 7, D and E). Moreover, cross-clade T cell responses were detected in spleen, lung, and NALT from Clec9A^OMNI^-boosted mice at 4 months after boost (Figure 7, F–H), and further subset analysis revealed clear CD4^+^ dominance in these mice (Supplemental Figure 8). These cellular immune responses also persisted at higher levels than those induced by BC mRNA vaccine booster (Figure 7, F–H), which displayed CD8^+^ skewing (Supplemental Figure 8). Greater proportions of CD4^+^ and CD8^+^ T cells with functional tissue-resident memory (T_RM_) phenotype were also detected in the lung and NALT from Clec9A^OMNI^-boosted mice compared with the BC mRNA–boosted group, which had proportions that were similar to naive controls (Figure 7, I and J).

Collectively, these results indicated that boosting nasally with Clec9A^OMNI^ induced cross-clade RBD-specific humoral and CD4-dominant T cell responses in both systemic and mucosal (respiratory) compartments. Furthermore, immune responses remained highly durable with the persistence of antibodies (IgG, IgA, and nAb), long-lived RBD-specific B cell subsets, T cell cytokine responses, and the establishment of respiratory mucosa-resident cellular memory.

### Nasal boosting with Clec9A^OMNI^ provided robust and sustained protection against SARS-CoV-2 challenge in the lower and upper airways.

**Figure 8:**
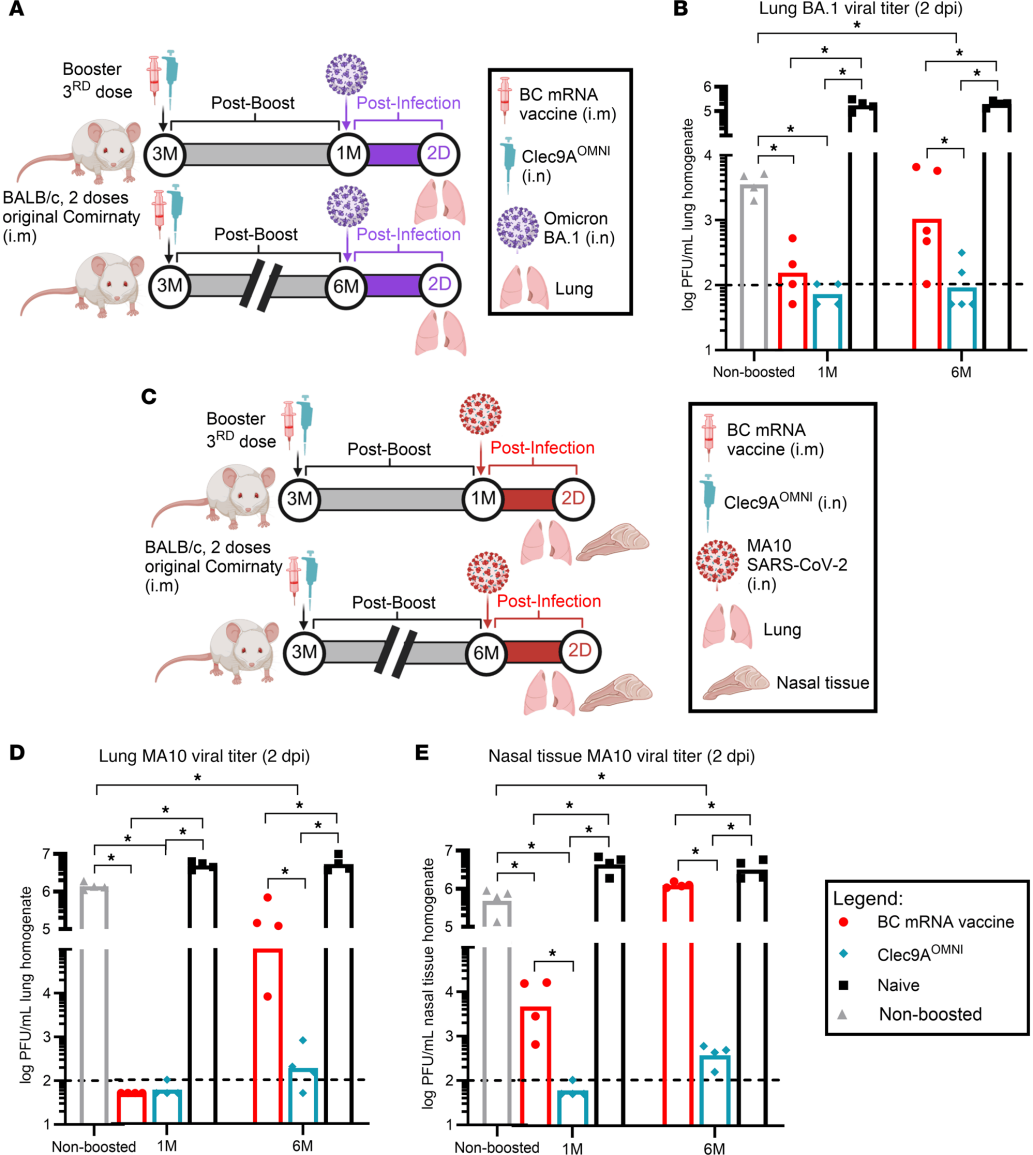
Protective efficacy of Clec9A^OMNI^ nasal booster versus BC mRNA vaccine systemic booster. (**A**) The 5- to six-week-old BALB/c mice were immunized with original Comirnaty mRNA vaccine as described in Figure 1. At 3 months after the second immunization dose, mice were boosted with either BC mRNA vaccine (0.05 μg; i.m.) or Clec9A^OMNI^ (4 μg Clec9A^XBB^ + 1 μg Clec9A^CoV1^ adjuvanted with 50 μg poly I:C; i.n.). Mice were challenged with Omicron BA.1 virus (10^6^ PFU; i.n.) at 1 and 6 months after boost. At 2 dpi, lungs were harvested and homogenized. (**B**) BA.1 viral titers in the lung homogenates were quantified via plaque assay. (**C**) The 5- to six-week-old BALB/c mice were immunized as described in **A**. Mice were challenged with MA10 SARS-CoV-2 virus (10^6^ PFU; i.n.) at 1 and 6 months after boost. At 2 dpi, the lung and nasal tissues were harvested and homogenized. (**D** and **E**) MA10 viral titers in the lung (**D**) and nasal (**E**) tissue homogenates were quantified via plaque assay. Data in **B**, **D**, and **E** are from 1 representative experiment performed twice with similar results; *n* = 4–5 per group/experiment. Symbols represent individual animals, and data shown are means. Statistical analysis: nonparametric 2-tailed Kruskal-Wallis test with Dunn’s multiple-comparison test. *P < 0.05.

Finally, we investigated the protective efficacy conferred by Clec9A^OMNI^ nasal booster immunization. At 1 and 6 months after boost, mice were challenged nasally with Omicron BA.1 virus, and 2 day postinfection (dpi) lung homogenate viral titers were measured (Figure 8A). Viral titers were largely undetectable in the lungs from both BC mRNA– and Clec9A^OMNI^-boosted mice when challenge was performed at 1 month after boost (Figure 8B). In contrast, when challenge was performed at 6 months after boost, substantial viral titers were detected in the lungs from BC mRNA–boosted mice, while viral titers remained at or below the detection limit in mice nasally boosted with Clec9A^OMNI^ (Figure 8B). These observations correlated well with the persistent systemic and mucosal humoral and cellular immune responses measured in the Clec9A^OMNI^-boosted group, while waning antibody responses and limited persistence of cellular subsets were clearly seen in BC mRNA–boosted mice (Figures 6 and 7).

To evaluate the protective efficacy of nasal Clec9A^OMNI^ booster immunization at the nasal mucosa, we next challenged mice with MA10, a mouse-adapted ancestral SARS-CoV-2 virus that can establish infection in the upper respiratory compartment from immune competent mice (unlike Omicron BA.1) (28) (Figure 8C). As seen with BA.1 challenge, MA10 viral titers measured at 1 month after challenge in the lung homogenates were below the detection limit in either boosted groups, but substantial viral titers were detected in the lungs from BC mRNA–boosted mice at 6 months after challenge while viral titers remained close to the detection limit in mice that received Clec9A^OMNI^ nasal booster (Figure 8D). In contrast, high (more than 3log_10_) viral titers were measured in the nasal tissue homogenates from BC mRNA–boosted mice at both 1 and 6 months after challenge, while the viral titers in Clec9A^OMNI^-boosted mice were remarkably lower and near the detection limit (Figure 8E).

Together, these results demonstrated that nasal booster immunization with Clec9A^OMNI^ conferred robust and sustained protection against SARS-CoV-2 challenge, which was superior to BC mRNA vaccine booster. Of particular importance, the robust protection against infection observed in the upper respiratory mucosa suggested that this vaccination approach may limit virus transmission and curb the evolution of escape variants.

## Discussion

Given that majority of the global population has developed some level of immunity to SARS-CoV-2, either through vaccination, infection, or both, prospective vaccine candidates targeting sarbecoviruses should broaden existing SARS-CoV-2 immunity and induce long-lasting responses. In this study, we showed that boosting simultaneously with Omicron XBB.1.5 and SARS-CoV-1 RBD using a Clec9A-targeting mAb (Clec9A^OMNI^) broadened the immune responses to clade 1a and 1b sarbecoviruses in Comirnaty mRNA–vaccinated mice. In Clec9A^OMNI^ -boosted animals, we detected a subset of B cells in the spleen and respiratory mucosa that were triple cross-reactive to RBD from ancestral SARS-CoV-2, XBB.1.5, and SARS-CoV-1, which strongly supported the production of broadly neutralizing antibodies, in addition to the production of clade-specific nAbs. Triple cross-reactive B cells were not detected in mice boosted with BC mRNA vaccine. Our findings supported that antigenic exposure to both sarbecovirus clades is required to elicit cross-clade humoral breadth, which is consistent with the low degree of conservation between RBD sequences (29), despite the presence of conserved B cell epitopes between clade 1a and 1b sarbecoviruses (30, 31). Although COVID-19 mRNA vaccines were shown to induce RBD-specific B cell clones that produced cross-clade nAb targeting these conserved epitopes (10, 31, 32), these B cell clones are extremely rare. Hence, boosting with Omicron-based mRNA vaccines is likely to limit the expansion of these cross-clade B cell clones and instead favors the recall of B cells that recognize epitopes that are shared within clade 1b and that are in greater abundance (30, 31). To avoid this immune imprinting phenomenon, it was proposed that boosting with SARS-CoV-1 RBD increases the selection stringency toward those rare cross-clade B cell clones and avoids the recall of clade 1b–specific B cell clones (33). Consistently, previous studies have reported the induction of pan-sarbecovirus nAb responses through sequential cross-clade vaccination strategies (34, 35). Likewise, SARS-CoV-2 infection or vaccination of individuals with preexisting SARS-CoV-1 immunity triggered cross-clade nAb responses (21).

While boosting with Clec9A^CoV1^ alone was sufficient to induce cross-clade humoral responses in Comirnaty mRNA–vaccinated mice, the RBD-specific T cell responses were mostly specific to SARS-CoV-1. On the other hand, boosting with Clec9A^XBB^ alone generated robust cellular responses against SARS-CoV-2 variants but weak T cell responses against SARS-CoV-1. Cross-clade T cell responses were only produced upon simultaneous boosting with the 2 Clec9A constructs (Clec9A^OMNI^). This finding suggested low T cell cross-reactivity between both sarbecovirus clades and indicated that the breadth of cellular immune responses induced by Clec9A^OMNI^ is likely mediated by a combination of de novo responses against clade 1a sarbecoviruses, coupled with recall of cross-reactive T cells from prior SARS-CoV-2 mRNA vaccination against clade 1b sarbecoviruses. Indeed, studies have reported low amino acid conservation between the immunodominant spike peptide pools of SARS-CoV-2 and SARS-CoV-1 (36). Therefore, to elicit both cross-clade nAb and T cell responses following prior Comirnaty mRNA vaccination, formulations that contain both clade 1a and 1b sarbecovirus RBD (e.g., chimeric antigen, mosaic nanoparticle, and multivalent cocktail vaccines) may represent better booster strategies (34, 37–39).

One of the unique features of the Clec9A-targeting strategy is the ability to induce exceptionally sustained immune responses, which have been associated with the persistence of GC B cells and Tfh cells (15, 16). Consistently, Clec9A^OMNI^ booster induced sustained antibody responses, which correlated with the persistence of RBD-specific Tfh cells, GC B cells, and increased differentiation into LLPCs. In contrast, boosting with BC mRNA vaccine failed to establish LLPCs in the BM, where the majority of RBD-specific ASCs displayed a non-LLPC phenotype, and in line with a previous report (40). The differentiation of GC B cells into LLPC is dependent on Tfh cell–derived cytokines, and costimulatory and regulatory signals (41, 42). We found that immunization with Clec9A^XBB^ induced a strong and persistent Tfh response, which was associated with sustained antibody titers, while immunization with Clec9A^CoV1^ induced broader responses, albeit a less potent and less durable Tfh cell response, which was associated with less persistent antibody titers. This observation suggested that the RBD antigenic sequence fused to the anti-Cle9A antibody influenced the immune responses. While the exact mechanism remains to be determined, variation in viral peptide and epitope sequences even by a single amino acid substitution greatly influenced the propensity of antigen-specific CD4^+^ T cells to polarize toward Tfh versus non-Tfh (e.g., T_H_1) lineages (43, 44). Clec9A^CoV1^ immunization triggered a strong CD4^+^ T_H_1 but poor Tfh response, whereas Clec9A^XBB^ immunization elicited a more balanced repertoire of T_H_1 and Tfh responses. Cytokines such as type 1 IFN, IL-6, and IL-12 drive the CD4^+^ T cell polarization toward a Tfh-biased phenotype (41, 45). Additionally, variation in antigenic sequences was reported to impact T cell receptor signal strength and may influence the polarization of CD4^+^ T helper subsets, whereby strong signaling was shown to promote terminal T_H_1 differentiation over Tfh (46, 47). Hence, the avidity of T cell interaction with their respective MHC-peptide complexes may differ, whereby Clec9A^CoV1^ may generate strong signaling that favored T_H_1 differentiation to the detriment of Tfh differentiation (36), thereby impairing downstream GC responses and skewing ASC differentiation toward non-LLPCs, leading to antibody waning. Recent studies have instead reported slower decline of spike and RBD antibodies in SARS-CoV-1 versus SARS-CoV-2 convalescent patients (30). However, the durability of cognate antibody responses upon vaccination versus infection are expected to be different. Consistently, SARS-CoV-2 convalescents displayed substantially more sustained spike- and RBD-specific antibody responses than mRNA vaccinees (48, 49). A possible explanation may be the broader T cell clonal diversity generated from infection, enabling spike- and RBD-specific B cells to receive additional costimulatory signals from Tfh cells specific to other viral antigens, thus promoting LLPC differentiation and survival, which would translate into more sustained antibody responses (50, 51).

Finally, this work reported the suitability of the nasal route for the delivery of Clec9A antibody constructs. We demonstrated that nasal Clec9A^OMNI^ booster in Comirnaty mRNA–vaccinated mice not only produced robust RBD-specific humoral and cellular responses at both the lower and upper respiratory mucosa but also potentiated the corresponding systemic immune responses. Potent cross-clade nAb and IgA responses, RBD-specific IgG^+^ and IgA^+^ LLPCs, and functional T_RM_ cells were measured in the lung and nasal compartments, which represent strong correlates of long-lived protection against infection and transmission (52–55). This was in sharp contrast to the limited induction of respiratory immunity seen upon BC mRNA i.m. booster, where IgG only was detected in the BALF and NLF, likely the result of serum IgG spillover (7). Inducing strong nAb response at mucosal sites is important to prevent viruses from establishing infection. IgA in particular is the first line of defense in blocking virus attachment and entry via transcytosis toward the apical surface of the mucosal epithelium (52–54, 56). However, mucosal IgG, which is largely localized at the basolateral surface, play a protective role in restricting virus replication and dissemination to adjacent tissues and the systemic circulation via direct neutralization or Fc receptor–mediated effector mechanisms (56–58). In addition, mucosal T_RM_ cells orchestrate local immune responses to facilitate efficient virus elimination by providing constant immune surveillance of the airway mucosa and initiating rapid recall responses upon cognate antigen encounter (52, 59, 60). Importantly, we found that nasal Clec9A^OMNI^ boost induced a subset of T_RM_ cells that were CD49a^+^, which has been associated with enhanced type 1 cytokine and cytolytic responses that have been shown to be critical for antiviral immunity (60–63). Therefore, effector responses from reactivated T_RM_ cells may potentially accelerate local viral clearance and offer early protection against infection, pulmonary disease, and mortality (59, 60, 62, 64).

In conclusion, although the relative role in protection of antibodies and T cells produced at the respiratory mucosa remains to be further investigated, we demonstrated that boosting Comirnaty mRNA–vaccinated mice with Clec9A^OMNI^ elicited sustained, robust, cross-clade, protective immune responses. Given that nAb represents a strong correlate of protection against infection, the ability to generate broadly neutralizing antibodies and cross-reactive B cells against both clade 1a and 1b sarbecoviruses makes Clec9A^OMNI^ an attractive pan-sarbecovirus vaccine candidate (4, 65). Furthermore, successful induction of robust and durable mucosal immunity in both the lower and upper respiratory compartments upon nasal boosting represents a game changer that is not only expected to limit virus infection and transmission, but may also curb the evolution of escape variants (66, 67).

As a plug-and-go platform, Clec9A-based constructs can be rapidly updated or engineered in response to newly emerging threats. Moreover, the availability of large-scale mAb manufacturing processes worldwide facilitates cost-effective production and rapid deployment of Clec9A-targeting vaccines during pandemics compared with other DC-targeting platforms (68, 69). Importantly, a human Clec9A-expressing cDC1 subset was found within numerous tissues and mucosae at constant numbers throughout the human lifespan (70). Our previous work in humanized mice and nonhuman primates (20, 71) supports that the Clec9A-targeting vaccine strategy can be readily translated to human populations. Hence, the Clec9A targeting technology holds great promise to tackle key shortcomings faced by current vaccine platforms.

## Methods

### Sex as a biological variable.

We used only female mice to reduce inter-mouse aggression during group housing for extended periods, which can cause wounding, stress, and inflammation that confound immunologic readouts following vaccination. While sex was not considered as a biological variable, generalization to males will require confirmation in future work using housing and handling conditions that mitigate male aggression.

### Mice.

5- to 6-week-old female BALB/c mice were purchased from InVivos and housed under specific pathogen-free conditions in individual ventilated cages.

### Cell lines and live SARS-CoV-2 virus.

CHO-K1 (ATCC, CCL-61) and CHO-Clec9A (CHO-K1 transfected in-house with plasmid encoding mouse Clec9A) cells were maintained in RPMI 1640 medium (Thermo Fisher Scientific, 22400-105) containing 5% FBS (Thermo Fisher Scientific, 10270-106), 10 mM HEPES (Thermo Fisher Scientific, 15630080), and 1% penicillin-streptomycin (Pen/Strep) (Thermo Fisher Scientific, 15140122) at 37°C and 5% CO_2_. Human lung alveolar A549 cells (ATCC, CRM-CCL-185) transduced in-house with lentivirus carrying human ACE2 gene in pFUGW vector (A549-ACE2) (provided by Wang Linfa, Duke-NUS Medical School, Singapore) were maintained in DMEM (Thermo Fisher Scientific, 11965-118) containing 10% FBS and 1% Pen/Strep at 37°C and 5% CO_2_.

The infectious clone (ic2019-nCoV MA10) of mouse-adapted SARS-CoV-2 MA10 virus (28) was obtained from BEI Resources (NR-55329), while Omicron BA.1 SARS-CoV-2 virus (GenBank accession OP643601) was provided by the National University of Singapore (NUS) Medicine Biosafety Level-3 Core Facility and Infectious Diseases Translational Research Programme, which was isolated from clinical samples obtained from the National Centre for Infectious Diseases and transferred to the NUS Medicine Pathogen Research Biosafety Level-2 lab for virus amplification and titration, as described previously (15). Briefly, MA10 SARS-CoV-2 and Omicron BA.1 viruses were grown in A549-ACE2 cells, and culture supernatants were harvested, clarified, and concentrated using Amicon 100 kDa filter unit (Merck Millipore, UFC910096) at 2 dpi. Plaque assay was then performed where virus-containing suspensions were serially diluted 10-fold and incubated with A549-ACE2 cells for 1 hour at 37°C and 5% CO_2_. The infected monolayers were overlaid with DMEM + 2% FBS medium containing 1.2% microcrystalline cellulose (Sigma-Aldrich, 435244). After 3 days incubation, cells were stained and fixed with 0.5% crystal violet containing 4% formaldehyde for plaque enumeration. Lung viral titer per milliliter (PFU/mL) was calculated as follows: [(plaque count)/(inoculum volume)] × dilution factor. Dashed lines in plotted data represent the limit of detection (LOD) at 2 log_10_, and samples below LOD were given an arbitrary value of half the LOD (1.7 log_10_).

### Clec9A-RBD constructs and mRNA vaccines.

The Clec9A-M2e construct was described previously (16, 17). To generate Clec9A^XBB^ and Clec9A^CoV1^ constructs, rat IgG2a mAb (10B4) specific against mouse Clec9A was genetically fused to 1 copy of Omicron XBB.1.5 or SARS-CoV-1 RBD (Supplemental Figure 9A) via a triple alanine linker at the C-terminal end of each heavy chain. Expression, purity, and binding of constructs to murine Clec9A were validated using previously described approaches (15). The Pfizer-BioNTech original Comirnaty mRNA vaccine based on Wuhan-Hu-1 (ancestral) SARS-CoV-2 spike protein and the BC mRNA vaccine based on ancestral and Omicron BA.4/5 spike proteins were both approved and obtained from the Ministry of Health Singapore.

### Mouse vaccination, challenge, and sample collection.

Mice were injected twice at 3-week intervals with 0.05 μg original Comirnaty in a volume of 20 μL via the i.m. route. Three months after the second dose, mice were boosted with 0.05 μg BC mRNA vaccine via the i.m. route in a volume of 20 μL, 3.75 μg Clec9A-M2e + 1 μg purified XBB.1.5 rRBD + 0.25 μg purified SARS-CoV-1 rRBD, supplemented with 50 μg poly I:C (Invivogen, vac-pic) via the i.n. route in a volume of 14 μL or with Clec9A-RBD constructs supplemented with 50 μg poly I:C, either via the s.c. or i.n. route in a volume of 100 or 40 μL, respectively. Unless otherwise specified in figure legends, a dose of 10 μg was used for Clec9A^XBB^ and Clec9A^CoV1^ s.c. immunization. For Clec9A^OMNI^, 8 μg Clec9A^XBB^ + 2 μg Clec9A^CoV1^ and 4 μg Clec9A^XBB^ + 1 μg Clec9A^CoV1^ were used for s.c. and i.n. immunization, respectively (unless otherwise specified in figure legends). Nonimmunized mice served as a negative control.

For challenge experiments, mice were infected i.n. with 10^6^ PFU MA10 SARS-CoV-2 and Omicron BA.1 in a volume of 20 μL at 1 and/or 6 months after booster immunization. Nonboosted mice were also included as a susceptibility control. Mice were euthanized at 2 dpi, and lung was harvested and placed in 2 mL tubes containing 1.4 mm ceramic beads (Omni International, 19-627D) and DMEM + 2% FBS. Nasal tissue was also harvested using an established protocol (72) and placed in similar tubes containing 1.4 mm ceramic beads and DMEM + 2% FBS. Whole lung and nasal tissues were homogenized for 1 minute at 4 m/s using Omni Bead Ruptor 12 (Omni International, 19-050A) and centrifuged for 1 minute at maximum speed to pellet tissue debris. The supernatants were collected as lung and nasal tissue homogenates and subjected to plaque assay for viral titer measurement as described above. Viral titers were expressed as log_10_ PFU/mL lung or nasal tissue homogenate.

Serum and BALF were collected as described previously (15). To obtain NLF, mice were euthanized, and a small incision was made at the trachea to allow insertion of a 22-gauge cannula into the nose. The nasal cavity was gently flushed with 1 mL chilled PBS supplemented with Halt protease inhibitor cocktail (1:100) (Thermo Fisher Scientific, 78429), and the fluid was collected in an Eppendorf tube placed beneath the mouse nostril. The recovered NLF was centrifuged at 400*g* for 5 minutes at 4°C to pellet cell debris before storing the supernatant at –80°C until further analysis.

Spleen, lung, femur, and tibia were harvested and processed into single-cell suspensions as described previously (15). NALT was harvested by dissecting the lower jaw and tongue to reveal the upper palate. After rinsing with chilled PBS, the upper palate was excised using a no. 11 scalpel blade and gently peeled back with fine forceps. The upper palate was minced into small pieces and digested with 0.1 mg/mL Liberase TL (Merck Millipore, 5401020001) in RPMI 1640 medium at 37°C and 5% CO_2_ for 45 minutes. Cells were passed through a 70 μm cell strainer and centrifuged and resuspended in complete RPMI (RPMI 1640 + 10% FBS + 1% Pen/Strep) to obtain a single-cell suspension of NALT.

### Recombinant proteins and peptides.

Purified recombinant ancestral SARS-CoV-2 and XBB.1.5 RBD were produced in-house as described previously (15). Purified recombinant SARS-CoV-1 RBD (SPD-S52H6) and biotinylated recombinant ancestral SARS-CoV-2 (SPD-C82E9), XBB.1.5 (SPD-C82Q3), and SARS-CoV-1 RBD (SPD-S82E3) were purchased from ACROBiosystems. RBD peptide pools from ancestral SARS-CoV-2; XBB.1.5, JN.1, clade 1b bat, and pangolin sarbecoviruses; RaTG13, Gx-P5L, and clade 1a sarbecoviruses; SARS-CoV-1; and WIV-1 were synthesized by Mimotopes. Each pool comprised 53 15-mer peptides overlapping by 11 residues and were reconstituted in DMSO (Sigma-Aldrich, 34869).

### ELISA.

ELISA plates (Sigma-Aldrich, M9410-1CS) were coated with 3 μg/mL of ancestral, XBB.1.5, or SARS-CoV-1 RBD diluted in PBS and incubated overnight at 4°C. For IgG measurement, mouse sera, BALF, and NLF were diluted 1:200, 1:20, and 1:10, respectively, followed by 2-fold serial dilutions in 1% BSA/PBS (Sigma-Aldrich, A7906). For IgA measurement, BALF and NLF were used neat. Plates were blocked with 5% BSA/PBS for 2 hours at room temperature (RT) and washed thrice with PBST (PBS and 0.05% Tween 20) before addition of diluted mouse sera, BALF, and NLF. After overnight incubation at 4°C, plates were washed thrice, and HRP-conjugated anti-mouse IgG (1:3,000) (Bio-Rad, 170-6516) and IgA (1:2,000) (Thermo Fisher Scientific, 62-6720) secondary antibodies diluted in 1% BSA/PBS were added and incubated overnight at 4°C. Subsequently, plates were washed thrice and developed using *o*-phenylenediamine dihydrochloride (OPD) (Sigma-Aldrich, P9187) and 3,30,5,50-tetramethylbenzidine (TMB) (Thermo Fisher Scientific, 00-4201-56) substrate for IgG and IgA detection, respectively. Plates were incubated for 10 minutes at RT in the dark, and the reaction was quenched with 1 M concentrated sulfuric acid. Absorbance was measured at 490 nm (OPD) and 450 nm (TMB) using a Sunrise absorbance microplate reader (Tecan). Anti-RBD IgG titers were determined by nonlinear regression as the reciprocal of the highest dilution, with A_490_ corresponding to 3 times the A_490_ of blank control wells. Dashed lines in plotted data represent the LOD at 200 (serum), 20 (BALF), and 10 (NLF). Anti-RBD IgA titers were expressed as A_450_ values following normalization against A_450_ of blank control wells.

### Multiplex surrogate virus neutralization test.

The multiplex surrogate virus neutralization test (sVNT) was established using the Luminex platform as described previously (1, 15). Briefly, RBDs from 22 sarbecoviruses (Supplemental Table 1) were enzymatically biotinylated and coated on MagPlex-Avidin microspheres. Mouse sera were diluted 1:10 and subjected to 3 4-fold serial dilutions. BALF was subjected to 3 2-fold serial dilutions, while NLF was undiluted. Equal volume of RBD-coated beads was preincubated with diluted mouse sera (final dilutions: 1:20, 1:80, 1:320, and 1:1,280), BALF (final dilutions: 1:2, 1:4, 1:8, and 1:16), and NLF (final dilution: 1:2) for 15 minutes at 37°C with agitation. Subsequently, PE-conjugated human ACE2 was added to each well and incubated for an additional 15 minutes at 37°C with agitation. After 2 washes with 1% BSA/PBS, the final readings were acquired using the MAGPIX system according to the manufacturer’s instructions. Percentage inhibition at each dilution was calculated as follows: [(MFI of 30 negative prepandemic samples – individual sample FI)/(MFI of 30 negative prepandemic samples)] × 100%. Serum and BALF nAb titers were reported as the reciprocal of the highest dilution that resulted in >50% inhibition (NT_50_), while NLF nAb titer was reported as percentage inhibition at 1:2 dilution, where a cutoff of 20% inhibition or greater was considered positive for nAbs against the sarbecovirus. Dashed lines in plotted data represent the LOD at 20 (serum) and 2 (BALF), or 20% cutoff for positive neutralization (NLF).

### Enzyme-linked immunosorbent spot assay.

Enzyme-linked immunosorbent spot (ELISpot) assay was performed to detect antigen-specific IgG and IgA ASCs. MultiScreen HTS-IP filter plates (Merck Millipore, MSIPS4W10) were prewetted with 35% ethanol for no longer than 1 minute, washed 4 times with PBS, and coated with 15 μg/mL purified anti-mouse IgG or IgA (Thermo Fisher Scientific, A16080, 62-6700) diluted in PBS. Following overnight incubation at 4°C, plates were washed 4 times with PBS and blocked with complete RPMI for 2 hours at RT. The blocking solution was discarded, and 10^6^ splenocytes, BM, lung, and NALT cells were added to each respective well and incubated for 40 hours at 37°C and 5% CO_2_. The cells were subsequently discarded, and plates were washed 4 times with PBST prior to the addition of 1 μg/mL biotinylated ancestral, XBB.1.5, and SARS-CoV-1 RBD diluted in 1% BSA/PBS. Biotinylated anti-mouse IgG and IgA (BioLegend, 405303, 407004) were included for detection of total IgG and IgA secretion as positive controls. After 2 hours incubation at RT, plates were washed 4 times with PBST, and HRP-conjugated streptavidin (SAv) (1:100) (BD Biosciences, 554066) diluted in 1% BSA/PBS was added and incubated for 1 hour at RT. Plates were washed 4 times with PBST, twice with PBS, and developed with AEC substrate (BD Biosciences, 551951) for 30 minutes before rinsing 10 times with deionized water. Spots were enumerated using the Mabtech IRIS ELISpot and FluoroSpot readers, and analysis was performed using Mabtech Apex software version 1.1.5.74. Data were expressed as spot forming units (SFUs) per 10^6^ cells.

### FluoroSpot.

Splenocytes and lung and NALT cells (5 × 10^5^) were restimulated for 40 hours at 37°C and 5% CO_2_ with 2.5 μg/mL ancestral SARS-CoV-2, XBB.1.5, JN.1, RaTG13, Gx-P5L, SARS-CoV-1, and WIV-1 RBD peptides, supplemented with 0.2 μg/mL kit-provided anti-mouse CD28. DMSO and 50 ng/mL PMA + 1 μg/mL ionomycin cocktail (BioLegend, 423302) were used as negative and positive controls, respectively. For analysis of CD4^+^ and CD8^+^ T cell responses, CD4^+^- and CD8^+^-enriched single-cell suspensions were prepared using mouse CD4 (L3T4) (Miltenyi Biotec, 130-117-043) and CD8α (Ly-2) (Miltenyi Biotec, 130-117-044) microbeads, according to the manufacturer’s protocol. Mouse IFN-γ/IL-2/TNF-α FluoroSpot (Mabtech, X-41A42B45W) was performed to evaluate antigen-specific cellular responses, according to the manufacturer’s protocol. Enumeration and analysis of spots were performed similarly as described for ELISpot, and data were expressed as SFUs per 5 × 10^5^ cells.

### Flow cytometry.

For antigen-specific B cell subsets, RBD-specific swIg^+^ and GC B cells were detected using biotinylated RBD antigens in combination with fluorophore-conjugated SAv. Biotinylated ancestral, XBB.1.5, and SARS-CoV-1 RBD were individually multimerized at a 4:1 molar ratio with SAv-BV421, SAv-PE, SAv-BV711, and SAv-APC (BD Biosciences, 563259, 554061, 563262, 554067). SAv-BV605 and SAv-BB515 (BD Biosciences, 563260, 564453) were used as decoy probes without biotinylated RBD to exclude cells with nonspecific binding to SAv. After multimerization, RBD probe master mix for swIg^+^ and GC B cell staining was created by mixing the respective fluorophore-conjugated RBD probes together (Supplemental Table 2) with 5 μM free d-biotin (Thermo Fisher Scientific, B20656) to minimize cross-reactivity between probes, diluted in a 1:3 mixture of Brilliant Stain buffer (BD Biosciences, 563794) and FACS buffer (2% FBS + 1 mM EDTA in PBS). Freshly isolated splenocytes and lung (10^6^ and 5 × 10^6^ for swIg^+^ and GC B cell analysis, respectively) and NALT cells (5 × 10^5^ for swIg^+^ B cell analysis) were incubated with anti-CD16/CD32 Fc block (BD Biosciences, 553142) and eFluor 780 Fixable Viability Dye (Thermo Fisher Scientific, 65-0865-14) diluted in FACS buffer (1:200 and 1:1,000, respectively) for 20 minutes at 4°C. Cells were washed with FACS buffer and stained with respective decoy probe (Supplemental Table 2) diluted in FACS buffer with 5 μM free d-biotin for 30 minutes at 4°C in the dark. Subsequently, cells were washed and stained with respective RBD probe master mix (Supplemental Table 2) for 1 hour at 4°C in the dark. Thereafter, cells were washed and stained with swIg^+^ or GC B cell surface marker antibodies (Supplemental Table 2) diluted in Brilliant Stain and FACS buffer for 30 minutes at 4°C in the dark.

For activation-induced marker (AIM^+^) Tfh cells, splenocytes and lung cells (2 × 10^6^) were restimulated with 2.5 μg/mL ancestral RBD peptides in the presence of 1 μg/mL purified anti-mouse CD40 and anti-mouse CD154-PE (1:100) (BioLegend, 102802, 106506) at 37°C and 5% CO_2_. DMSO and PMA + ionomycin cocktail were included as negative and positive controls, respectively, similarly as above. After overnight incubation, cells were washed and incubated with anti-CD16/CD32 Fc block and eFluor780 Fixable Viability Dye, similarly as above. Thereafter, cells were washed and stained with AIM^+^ Tfh cell surface marker antibodies (Supplemental Table 2), similarly as above.

For T_RM_ cells, freshly isolated lung (10^6^) and NALT (5 × 10^5^) cells were incubated with anti-CD16/CD32 Fc block and eFluor780 fixable viability dye, similarly as above. Cells were washed and stained with T_RM_ cell surface marker antibodies (Supplemental Table 2), similarly as above.

### Data acquisition and analysis.

Cells were washed and resuspended in FACS buffer after the final staining step prior to data acquisition on the LSRFortessa II X-20 analyzer (BD Biosciences). UltraComp eBeads (Thermo Fisher Scientific, 01-222-42) were used for single-color compensation controls. Gating strategies and representative analysis results for antigen-specific B cell subsets and AIM^+^ Tfh and T_RM_ cells can be found in Supplemental Figures 10–15.

### FACS sorting of LLPC and non-LLPC ASC subsets.

Freshly isolated BM (6–8 × 10^7^) and lung (3–4 × 10^7^) cells were incubated with anti-CD16/CD32 Fc block and eFluor780 fixable viability dye, similarly as above. Cells were washed and stained with ASC subset surface marker antibodies (Supplemental Table 2) diluted in Brilliant Stain and FACS buffer for 30 minutes at 4°C. Thereafter, cells were washed, resuspended in FACS buffer, and sorted for LLPC and non-LLPC subsets using the FACSAria Fusion Cell Sorter (BD Biosciences) and gating strategy indicated in Supplemental Figure 12. Total and anti-RBD IgG and IgA secretion from each ASC subset was assessed by B cell ELISpot as described above, which quantified frequency of total and anti-RBD IgG and IgA-secreting cells. The numbers of input ASCs from each subset were 1,500–2,500 for total IgG and IgA detection and 15,000–25,000 for anti-RBD IgG and IgA detection. Data were normalized and expressed as percent anti-RBD IgG or IgA ASC out of total IgG or IgA ASC for each subset, which was calculated as follows: (SFU_RBD_ _IgG_ _or_ _IgA_)/(SFU_Total_ _IgG_ _or_ _IgA_) × 100%.

### Statistics.

All data were plotted and analyzed using GraphPad Prism version 10. *P* values < 0.05 were considered statistically significant. Details about sample size, descriptive statistics, and statistical tests used are outlined in the figure legends. In brief, nonparametric 2-tailed Mann-Whitney test (2 groups) and Kruskal-Wallis test (more than 2 groups) with Dunn’s multiple-comparison test were used for unpaired group analysis, while Wilcoxon’s matched-pairs signed rank test (2 groups) and Friedman’s test with Dunn’s multiple-comparison test (more than 2 groups) were used for paired group analysis.

### Study approval.

The in vivo experiments described were approved by the Institutional Animal Care and Use Committee of NUS under protocol numbers R20-0392 and R24-0190 and were performed in accordance with the guidelines of the National Advisory Committee for Laboratory Animal Research. The NUS animal facilities are AAALAAC accredited and licensed by the regulatory body Agri-Food and Veterinary Authority of Singapore. All efforts were made to minimize animal suffering.

### Data availability.

Values for all data points in graphs are reported in the Supporting Data Values file.

## Author contributions

NYZC, MHL, and SA conceptualized the experiments and analyzed the data. NYZC, WCY, PST, XQ, KMT, KP, WYT, and SYYM performed the experiments. PAM and CWT provided reagents and inputs. NYZC wrote the original draft of the manuscript. MHL and SA reviewed and edited the manuscript.

## Funding support

- Programme for Research in Epidemic Preparedness and Response grant PREPARE-CS1-2022-002 to SA.

## Supplementary Material

Supplemental data

Supporting data values

## Version 1 01/15/2026

In-Press Preview

## Version 2 03/16/2026

Electronic publication
