# Supplementary material for: Intranasal DC-targeting vaccine booster elicits durable and cross-clade protective immunity against sarbecoviruses in mice
Source: J Clin Invest. 2026 Jan 15;136(6):e195784. doi: 10.1172/JCI195784 (PMC12987630; doi:10.1172/JCI195784)
Supplement: Supplemental data [file jci-136-195784-s114.pdf]

SUPPLEMENTAL MATERIALS

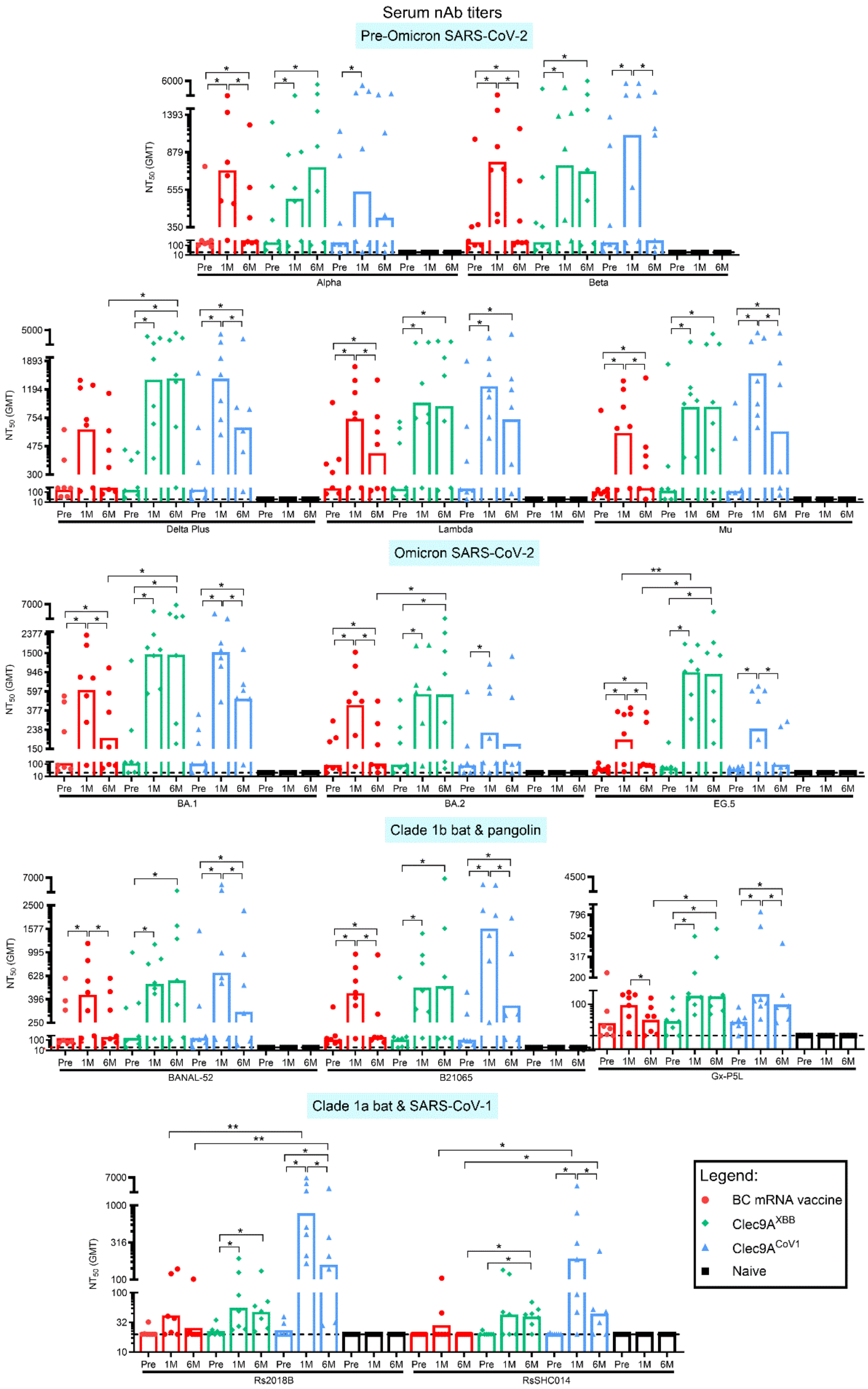

Figure S1. Serum neutralizing antibody titers upon systemic booster with Clec9A^{XBB}, Clec9A^{CoV1} or bivalent Comirnaty (BC) mRNA vaccine. Five to six-week-old BALB/c mice were immunized twice three weeks apart (0.05 µg per dose; i.m) with Pfizer-BioNTech original Comirnaty mRNA vaccine. Three months after the last immunization dose, mice were boosted either with Pfizer-BioNTech BA.4/5 bivalent Comirnaty (BC) mRNA vaccine (0.05 µg; i.m), Clec9A^{XBB} (10 µg adjuvanted with 50 µg poly I:C; s.c) or Clec9A^{CoV1} (10 µg adjuvanted with 50 µg poly I:C; s.c). A control group of non-immunized mice (naïve) was also included for baseline. The serum nAb titers against 13 sarbecoviruses representative of clades 1b and 1a at pre-boost, one- and six months post-boost were determined by multiplex sVNT. Data are from one representative experiment performed twice with similar results, n = 6-7 per group/experiment. Symbols represent individual animals and data shown are geometric means. Statistical analysis: Non-parametric two-tailed Kruskal Walis test with Dunn's multiple-comparison test and Friedman test with Dunn's multiple-comparison test. $^{*}p < 0.05$, $^{**}p < 0.01$.

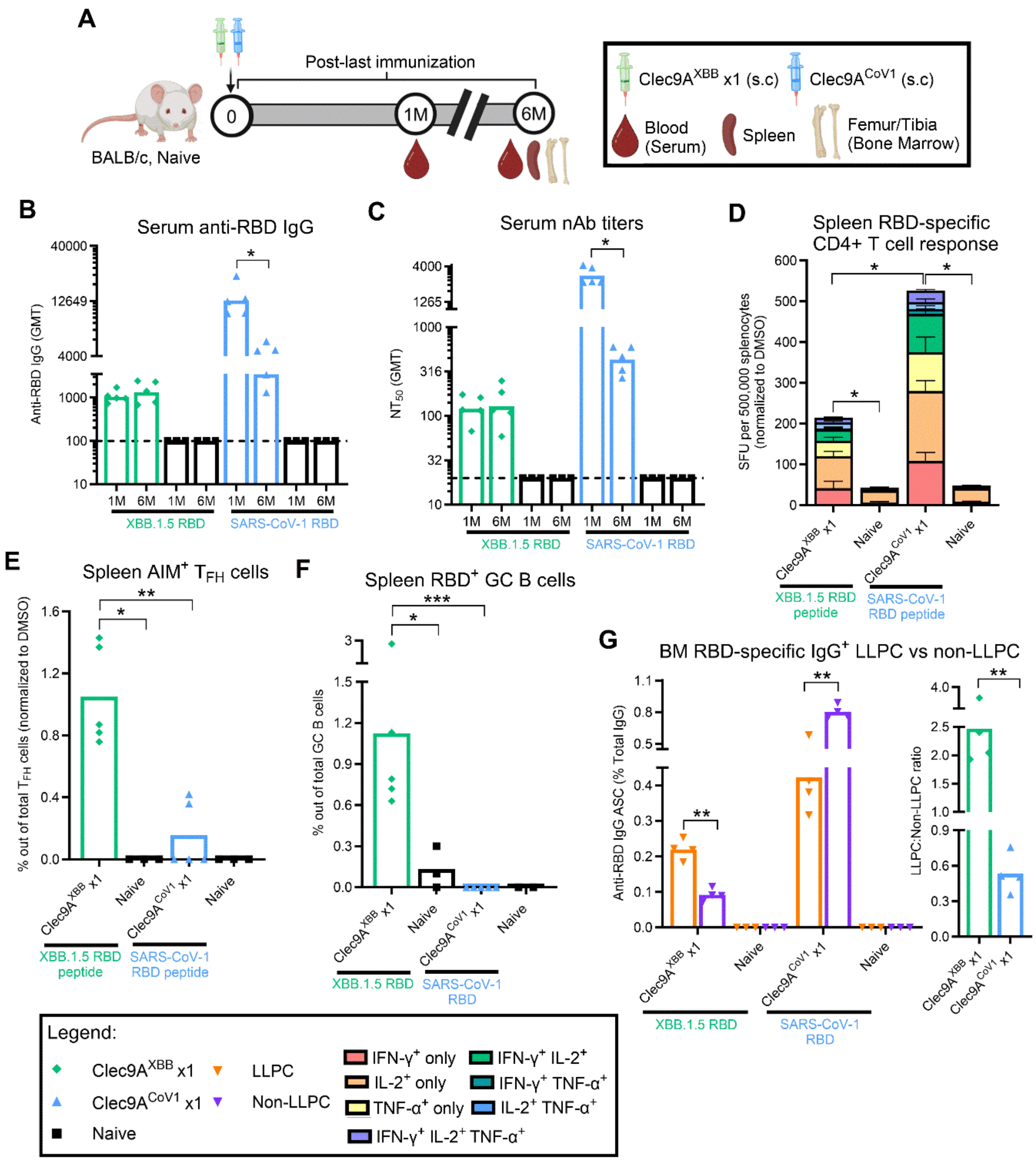

Figure S2. Durability of immune responses upon single shot immunization with Clec9A^{XBB} or Clec9A^{CoV1} in naïve mice. A) Five to six-week-old BALB/c mice were immunized with a single dose (2 µg adjuvanted with 50 µg poly I:C; s.c) of Clec9A^{XBB} or Clec9A^{CoV1}. **(B, C)** Blood was collected one- and six months after immunization. **(B)** Serum anti-RBD IgG titers against XBB.1.5 and SARS-CoV-1 RBD 1 at one- and six months post-immunization were determined by ELISA. **(C)** Serum nAb titers against

XBB.1.5 and SARS-CoV-1 at one- and six months post-immunization were determined by sVNT. **(D-G)** Mice were euthanized at six months post-immunization, and spleen and BM from femur/tibia were harvested. **(D)** Frequency of IFN-γ, IL-2 and/or TNF-α secreting $CD4^+$-enriched splenocytes at six months post-immunization was determined by FluoroSPOT upon re-stimulation with XBB.1.5 and SARS-CoV-1 RBD peptides. **(E, F)** Percentage of **(E)** AIM^+ T_{FH} and **(F)** RBD^+ GC B cells in spleen at six months post-immunization was determined by flow cytometry. **(G)** Frequency of BM RBD-specific IgG^+ LLPC and non-LLPC (normalized to total IgG) at six months post-immunization was determined by B cell ELISPOT. **(B-G)** Data are from one representative experiment performed twice with similar results, n = 4-5 per group/experiment. **(B, C, E-G)** Symbols represent individual animals and data shown are **(B, C)** geometric means and **(D-G)** means ± **(D)** SD. Statistical analysis: Non-parametric two-tailed **(B, C)** Wilcoxon matched-pairs signed rank test and **(D-G)** Mann-Whitney test. *$p < 0.05$, **$p < 0.01$, ***$p < 0.001$.

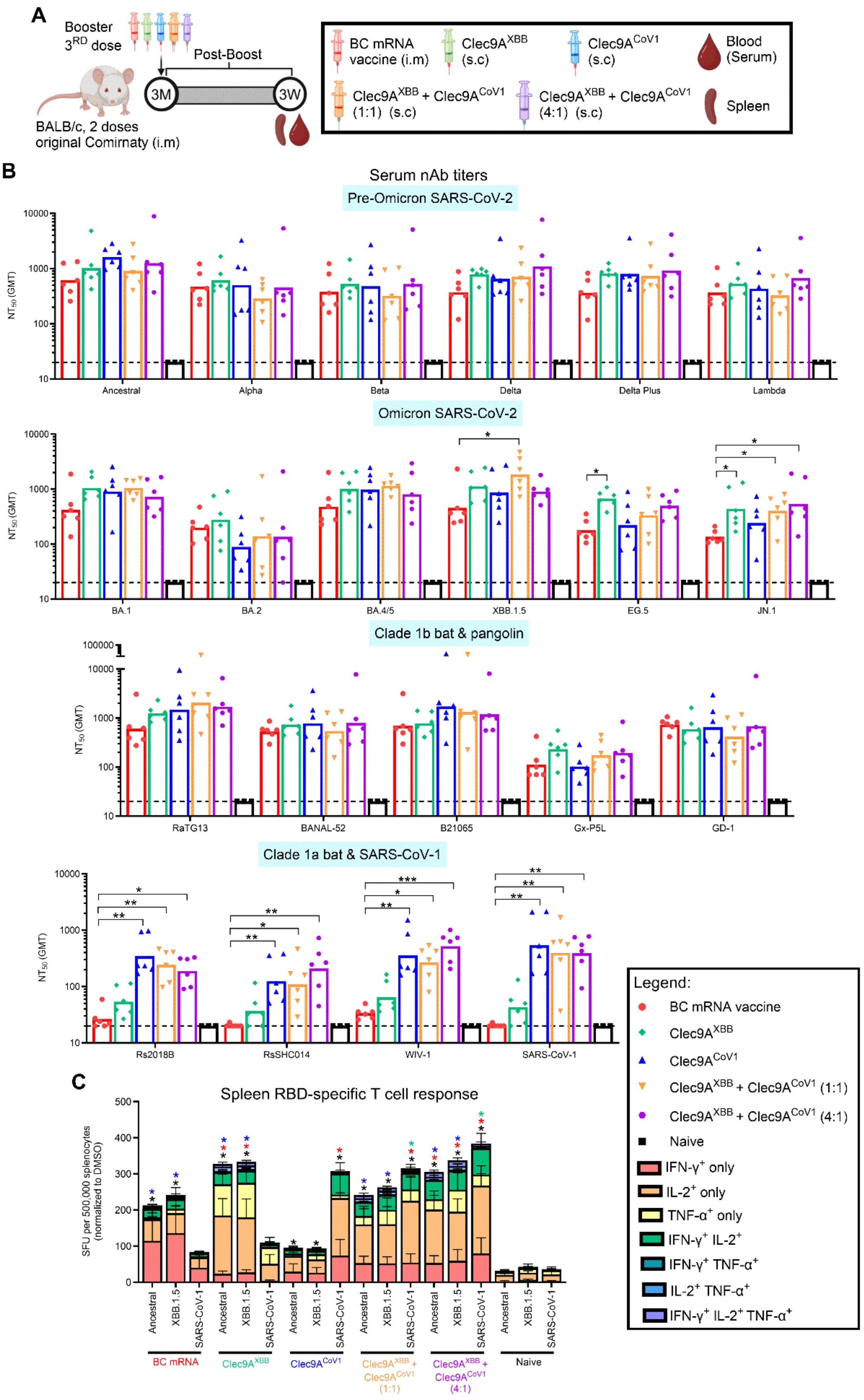

A
Booster 3RD dose
Post-Boost
3M
3W
BALB/c, 2 doses original Comirnaty (i.m)
BC mRNA vaccine (i.m)
Clec9AXBB (s.c)
Clec9ACoV1 (s.c)
Blood (Serum)
Clec9AXBB + Clec9ACoV1 (1:1) (s.c)
Clec9AXBB + Clec9ACoV1 (4:1) (s.c)
Spleen
B
Serum nAb titers
Pre-Omicron SARS-CoV-2
NT50 (GMT)
Ancestral
Alpha
Beta
Delta
Delta Plus
Lambda
Omicron SARS-CoV-2
BA.1
BA.2
BA.4/5
XBB.1.5
EG.5
JN.1
Clade 1b bat & pangolin
RaTG13
BANAL-52
B21065
Gx-P5L
GD-1
Clade 1a bat & SARS-CoV-1
Rs2018B
RsSHC014
WIV-1
SARS-CoV-1
C
Spleen RBD-specific T cell response
SFU per 500,000 splenocytes (normalized to DMSO)
Ancestral
XBB.1.5
SARS-CoV-1
BC mRNA
Clec9AXBB
Clec9ACoV1
Clec9AXBB + Clec9ACoV1 (1:1)
Clec9AXBB + Clec9ACoV1 (4:1)
Naive
Legend:
BC mRNA vaccine
Clec9AXBB
Clec9ACoV1
Clec9AXBB + Clec9ACoV1 (1:1)
Clec9AXBB + Clec9ACoV1 (4:1)
Naive
IFN-γ+ only
IL-2+ only
TNF-α+ only
IFN-γ+ IL-2+
IFN-γ+ TNF-α+
IL-2+ TNF-α+
IFN-γ+ IL-2+ TNF-α+

Figure S3. Dose optimization of [Clec9A^{XBB} + Clec9A^{CoV1}] booster. (A) Five to six-week-old BALB/c mice were immunized twice three weeks apart (0.05 µg per dose; i.m) with original Comirnaty mRNA vaccine. Three months after the second immunizing dose, mice were boosted with either BC mRNA vaccine (0.05 µg; i.m), Clec9A^{XBB} (10 µg), Clec9A^{CoV1} (10 µg), [Clec9A^{XBB} + Clec9A^{CoV1}] (5 µg + 5 µg) or [Clec9A^{XBB} + Clec9A^{CoV1}] (8 µg + 2 µg). All Clec9A-based formulations were adjuvanted with 50 µg poly I:C and administered s.c. At three weeks post-boost, blood was collected, and mice were euthanized to harvest spleen. **(B)** Serum nAb titers against 21 sarbecoviruses from clades 1a and 1b at three weeks post-boost was determined by multiplex sVNT. **(C)** Frequency of IFN-γ, IL-2 and/or TNF-α secreting splenocytes at three weeks post-boost was determined by FluoroSPOT upon re-stimulation with ancestral SARS-CoV-2, XBB.1.5 and SARS-CoV-1 RBD peptides. **(B, C)** Data are from one representative experiment performed twice with similar results, n = 5-6 per group/experiment. **(B)** Symbols represent individual animals and data shown are **(B)** geometric means and **(C)** means ± SD. Statistical analysis: Non-parametric two-tailed Kruskal Walis test with Dunn's multiple-comparison test. *$p < 0.05$, **$p < 0.01$, ***$p < 0.001$. **(C)** Asterisk colors represent statistical significance between corresponding groups.

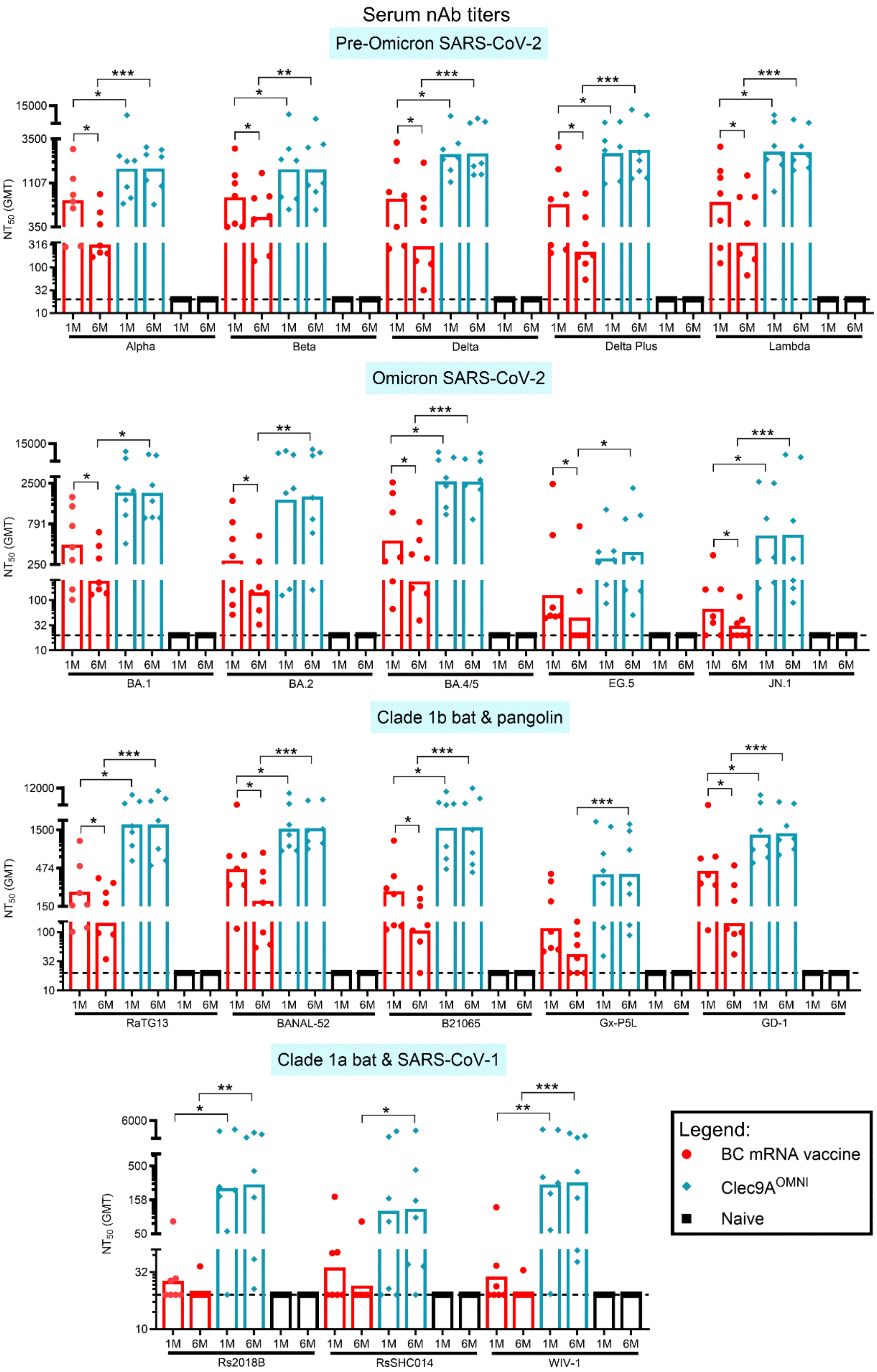

Serum nAb titers
Pre-Omicron SARS-CoV-2
Omicron SARS-CoV-2
Clade 1b bat & pangolin
Clade 1a bat & SARS-CoV-1
NT50 (GMT)
1M
6M
Alpha
Beta
Delta
Delta Plus
Lambda
BA.1
BA.2
BA.4/5
EG.5
JN.1
RaTG13
BANAL-52
B21065
Gx-P5L
GD-1
Rs2018B
RsSHC014
WIV-1
Legend:
BC mRNA vaccine
Clec9AOMNI
Naive

Figure S4. Serum neutralizing antibody responses upon systemic booster with Clec9A^{OMNI} versus BC mRNA vaccine. Five to six-week-old BALB/c mice were immunized twice three weeks apart (0.05 µg per dose; i.m) with original Comirnaty mRNA vaccine. Three months after the second immunization dose, mice were boosted with either BC mRNA vaccine (0.05 µg; i.m), or Clec9A^{OMNI} (8 µg Clec9A^{XBB} + 2 µg Clec9A^{CoV1} adjuvanted with 50 µg poly I:C; s.c). Serum nAb titers against 18 sarbecoviruses from clades 1a and 1b at one- and six months post-boost were determined by multiplex sVNT. Data are from one representative experiment performed twice with similar results, n = 5-6 per group/experiment. Symbols represent individual animals and data shown are geometric means. Statistical analysis: Non-parametric two-tailed Mann-Whitney test, and Friedman test with Dunn's multiple-comparison test. $^{*}p < 0.05$, $^{**}p < 0.01$, $^{***}p < 0.001$.

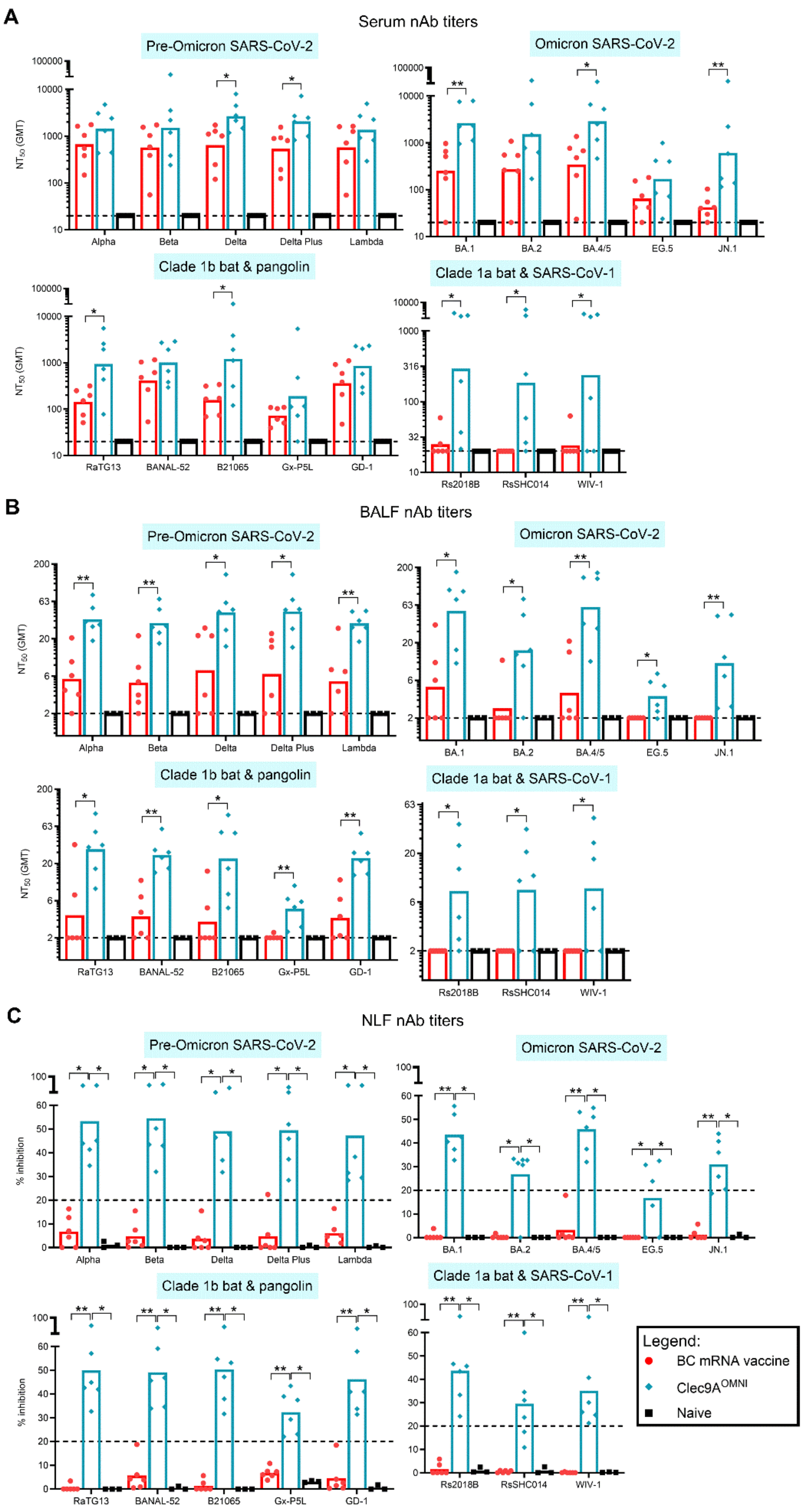

A
Serum nAb titers
Pre-Omicron SARS-CoV-2
Omicron SARS-CoV-2
NT50 (GMT)
Alpha
Beta
Delta
Delta Plus
Lambda
BA.1
BA.2
BA.4/5
EG.5
JN.1
Clade 1b bat & pangolin
Clade 1a bat & SARS-CoV-1
RaTG13
BANAL-52
B21065
Gx-P5L
GD-1
Rs2018B
RsSHC014
WIV-1
B
BALF nAb titers
C
NLF nAb titers
% inhibition
Legend:
BC mRNA vaccine
Clec9A OMNI
Naive

Figure S5. Neutralizing antibody responses upon nasal booster with Clec9A^{OMNI} versus systemic booster with BC mRNA vaccine. Five to six-week-old BALB/c mice were immunized twice three weeks apart with original Comirnaty mRNA vaccine (0.05 µg per dose; i.m). Three months after the second immunization dose, mice were boosted with either BC mRNA vaccine (0.05 µg; i.m), or Clec9A^{OMNI} (4 µg Clec9A^{XBB} + 1 µg Clec9A^{CoV1} adjuvanted with 50 µg poly I:C; i.n). **(A)** Serum, **(B)** BALF and **(C)** NLF nAb titers against 18 sarbecoviruses from clades 1a and 1b at one month post-boost were determined by multiplex sVNT. **(A-C)** Data are from one representative experiment performed twice with similar results, n = 5-6 per group/experiment. Symbols represent individual animals and data shown are **(A, B)** geometric means and **(C)** means. Statistical analysis: Non-parametric two-tailed **(A, B)** Mann-Whitney test and **(C)** Kruskal Walis test with Dunn's multiple-comparison test. *$p < 0.05$, **$p < 0.01$.

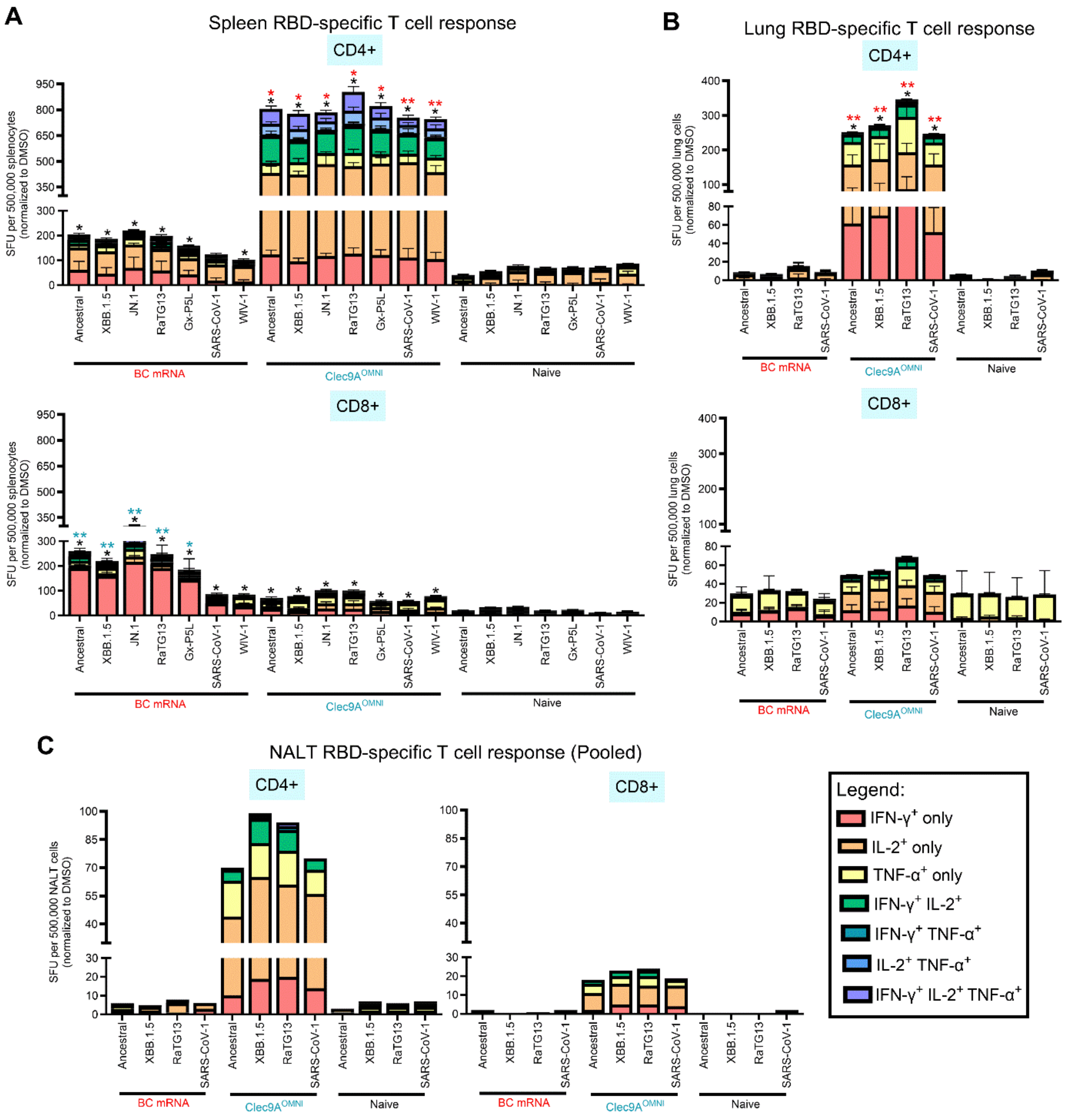

Figure S6. CD4^{+} and CD8^{+} T cell responses upon nasal booster with Clec9A^{OMNI} versus systemic booster with BC mRNA vaccine. Five to six-week-old BALB/c mice were immunized twice three weeks apart with original Comirnaty mRNA vaccine (0.05 µg per dose; i.m). Three months after the second immunization dose, mice were boosted with either BC mRNA vaccine (0.05 µg; i.m), or Clec9A^{OMNI} (4 µg Clec9A^{XBB} + 1 µg Clec9A^{CoV1} adjuvanted with 50 µg poly I:C; i.n). **(A-C)** At two weeks post-boost, mice were euthanized and the frequencies of IFN-γ, IL-2 and/or TNF-α secreting CD4^{+} and CD8^{+} subsets from

(A) spleen, **(B)** lungs, and **(C)** NALT were determined by FluoroSPOT upon restimulation with ancestral SARS-CoV-2, XBB.1.5, JN.1, RaTG13, Gx-P5L, SARS-CoV-1 and WIV-1 RBD peptides. **(A-C)** Data are from one representative experiment performed twice with similar results, n = 5 per group/experiment. Data shown are means ± **(A, B)** SD. Statistical analysis: **(A, B)** Non-parametric two-tailed Kruskal Walis test with Dunn's multiple-comparison test. *$p < 0.05$, **$p < 0.01$. Asterisk colors represent statistical significance between corresponding groups.

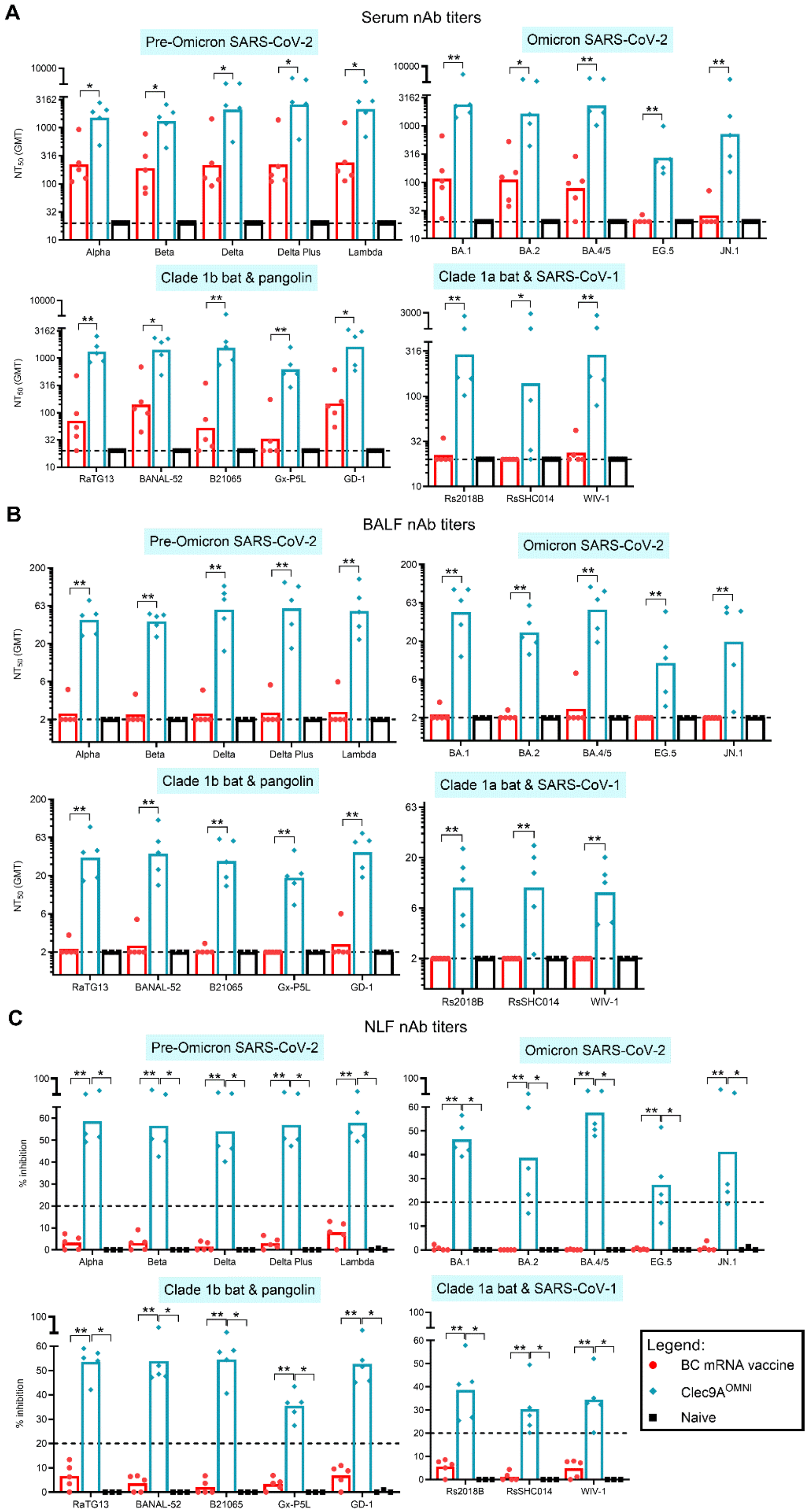
A
Serum nAb titers
Pre-Omicron SARS-CoV-2
Omicron SARS-CoV-2
Clade 1b bat & pangolin
Clade 1a bat & SARS-CoV-1
NT50 (GMT)
Alpha
Beta
Delta
Delta Plus
Lambda
BA.1
BA.2
BA.4/5
EG.5
JN.1
RaTG13
BANAL-52
B21065
Gx-P5L
GD-1
Rs2018B
RsSHC014
WIV-1
B
BALF nAb titers
C
NLF nAb titers
% inhibition
Legend:
BC mRNA vaccine
Clec9AOMNI
Naive

Figure S7. Long-term neutralizing antibody responses upon nasal booster with Clec9A^{OMNI} versus systemic booster with BC mRNA vaccine. Five to six-week-old BALB/c mice were immunized twice three weeks apart with original Comirnaty mRNA vaccine (0.05 µg per dose; i.m). Three months after the second immunization dose, mice were boosted with either BC mRNA vaccine (0.05 µg; i.m), or Clec9A^{OMNI} (4 µg Clec9A^{XBB} + 1 µg Clec9A^{CoV1} adjuvanted with 50 µg poly I:C; i.n). **(A)** Serum, **(B)** BALF and **(C)** NLF nAb titers against 18 sarbecoviruses from clades 1a and 1b at six months post-boost were determined by multiplex sVNT. **(A-C)** Data are from one representative experiment performed twice with similar results, n = 5-6 per group/experiment. Symbols represent individual animals and data shown are **(A, B)** geometric means and **(C)** means. Statistical analysis: Non-parametric two-tailed **(A, B)** Mann-Whitney test and **(C)** Kruskal Walis test with Dunn's multiple-comparison test. $*p < 0.05$, $**p < 0.01$.

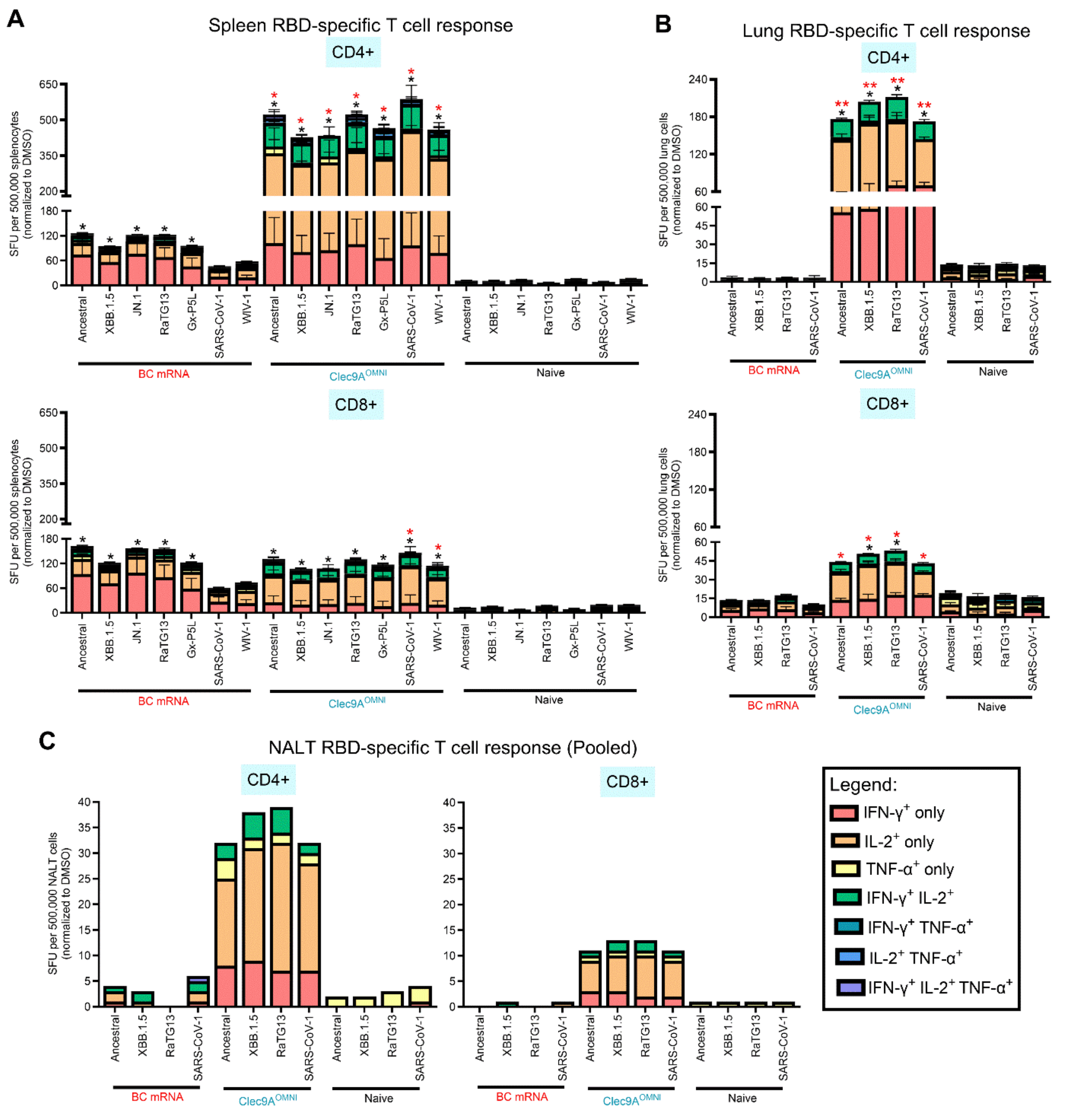

Figure S8. Long-term CD4^{+} and CD8^{+} T cell responses upon nasal booster with Clec9A^{OMNI} versus systemic booster with BC mRNA vaccine. Five to six-week-old BALB/c mice were immunized twice three weeks apart with original Comirnaty mRNA vaccine (0.05 µg per dose; i.m). Three months after the second immunization dose, mice were boosted with either BC mRNA vaccine (0.05 µg; i.m), or Clec9A^{OMNI} (4 µg Clec9A^{XBB} + 1 µg Clec9A^{CoV1} adjuvanted with 50 µg poly I:C; i.n). **(A-C)** Frequencies of IFN-γ, IL-2 and/or TNF-α secreting CD4^{+} and CD8^{+} subsets in **(A)** spleen, **(B)** lungs, and **(C)** NALT were determined

at four months post-boost by FluoroSPOT upon restimulation with ancestral SARS-CoV-2, XBB.1.5, JN.1, RaTG13, Gx-P5L, SARS-CoV-1 and WIV-1 RBD peptides. **(A-C)** Data are from one representative experiment performed twice with similar results, n = 5 per group/experiment. Data shown are means ± **(A, B)** SD. Statistical analysis: **(A, B)** Non-parametric two-tailed Kruskal Walis test with Dunn's multiple-comparison test. *p < 0.05, **p < 0.01. Asterisk colors represent statistical significance between corresponding groups.

A

```
XBB.1.5 RBD       1     KSFTVEKGIYQTSNFRVQPTESIVRFPNITNLCPFHEVFNATTFASVYAWNRKRISNCVA   60
                        KSF ++KGIYQTSNFRV P+   +VRFPNITNLCPF EVFNAT F SVYAW RK+ISNCVA
SARS-CoV-1 RBD    1     KSFEIDKGIYQTSNFRVVPSGDVVRFPNITNLCPFGEVFNATKFPSVYAWERKKISNCVA   60

XBB.1.5 RBD       61    DYSVIYNFAPFFAFKCYGVSPTKLNDLCFTNVYADSFVIRGNEVSQIAPGQTGNIADYNY   120
                        DYSV+YN    F  FKCYGVS TKLNDLCF+NVYADSFV++G++V QIAPGQTG IADYNY
SARS-CoV-1 RBD    61    DYSVLYNSTFFSTFKCYGVSATKLNDLCFSNVYADSFVVKGDDVRQIAPGQTGVIADYNY   120

XBB.1.5 RBD       121   KLPDDFTGCVIAWNSNKLDSKPSGNYNYLYRLFRKSKLKPFERDISTEIYQAGNKPCNGV   180
                        KLPDDF GCV+AWN+   +D+   +GNYNY YR  R  KL+PFERDIS    +    KPC
SARS-CoV-1 RBD    121   KLPDDFMGCVLAWNTRNIDATSTGNYNYKYRYLRHGKLRPFERDISNVPFSPDGKPCTPP   180

XBB.1.5 RBD       181   AGPNCYSPLQSYGFRPTYGVGHQPYRVVVLSFELLHAPATVCGPKKSTNLVKNKCVNFNF   240
                        A  NCY PL   YGF   T G+G+QPYRVVVLSFELL+APATVCGPK ST+L+KN+CVNFNF
SARS-CoV-1 RBD    181   A-LNCYWPLNDYGFYTTTGIGYQPYRVVVLSFELLNAPATVCGPKLSTDLIKNQCVNFNF   239

XBB.1.5 RBD       241   NGLTGTG   247
                        NGLTGTG
SARS-CoV-1 RBD    240   NGLTGTG   246
```

B

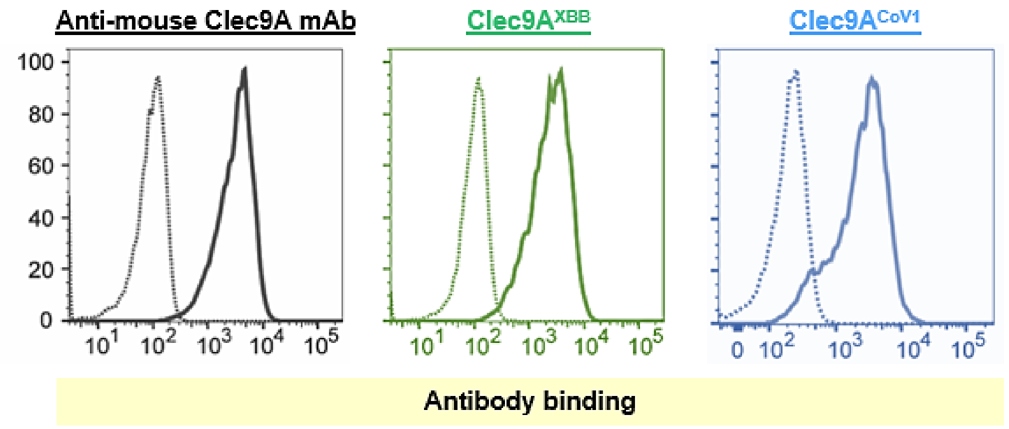

Figure S9. Antigenic sequence and validation of Clec9A-RBD constructs. (A) BLAST amino acid sequence alignment for RBD antigens (XBB.1.5 and SARS-CoV-1) used for expression of Clec9A-RBD constructs (Clec9A^{XBB} and Clec9A^{CoV1}). **(B)** Binding of Clec9A-RBD constructs to mouse Clec9A was verified by flow cytometry. CHO-K1 (dotted lines) or CHO-Clec9A cells (solid lines) were incubated with control anti-mouse Clec9A mAb (black, left panel), purified Clec9A^{XBB} construct (green, middle panel) or

Clec9A^{CoV1} construct (blue, right panel). Binding was detected with PE-conjugated anti-rat Ig and dead cells were excluded by forward/side scatter and live/dead staining.

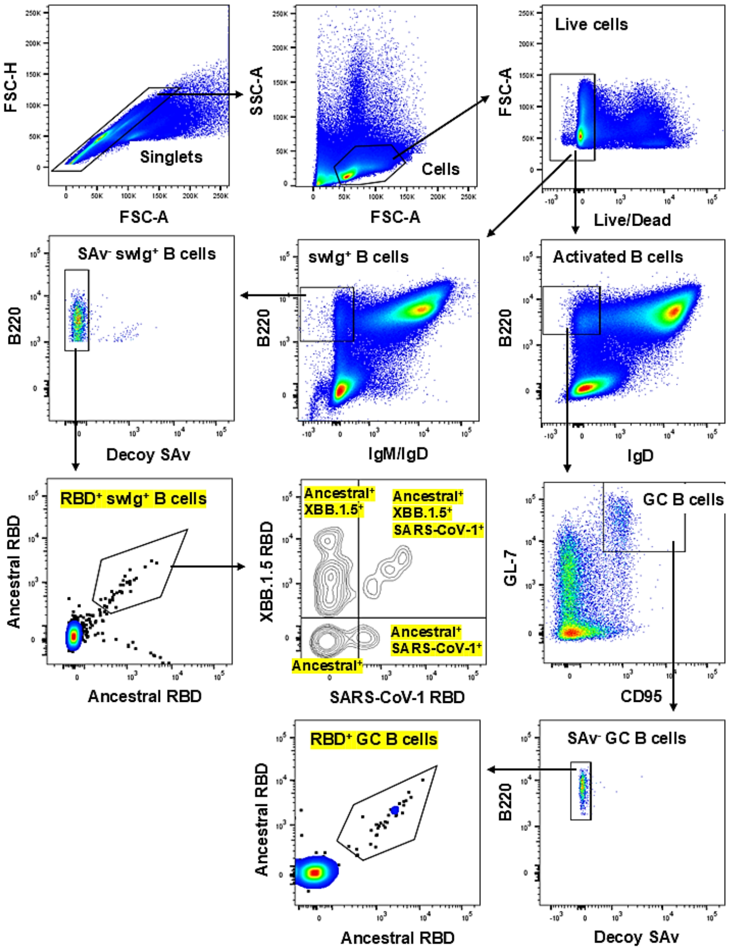

Figure S10. Gating strategy to identify antigen-specific swIg$^+$ and GC B cell subsets. Single cells were first identified via FSC-H/FSC-A and SSC-A/FSC-A. Dead cells were excluded with eFluor780 Fixable Viability Dye, and swIg$^+$ (B220$^+$ IgD$^-$ IgM$^-$) and activated (B220$^+$ IgD$^-$) B cells were identified from live cell population (Live/Dead$^-$). For the former, swIg$^+$ B cells that bind non-specifically to SAv were excluded using decoy SAv probe, and RBD-specific swIg$^+$ B cells were identified from the SAv$^-$ swIg$^+$ B cell population via a double discrimination gate where cells must be double positive for both ancestral RBD-BV421 and -PE to be considered as antigen-specific. Cross-reactivity to XBB.1.5 and SARS-CoV-1 RBD were further determined from the gated total RBD$^+$ swIg$^+$ B cell population. For the latter, GC B cells (GL-7$^+$ CD95$^+$) were identified from the activated B cell population, and those binding non-specifically to SAv were excluded using decoy SAv probe. RBD-specific GC B cells were subsequently identified from the SAV$^-$ GC B cell population via double discrimination gating.

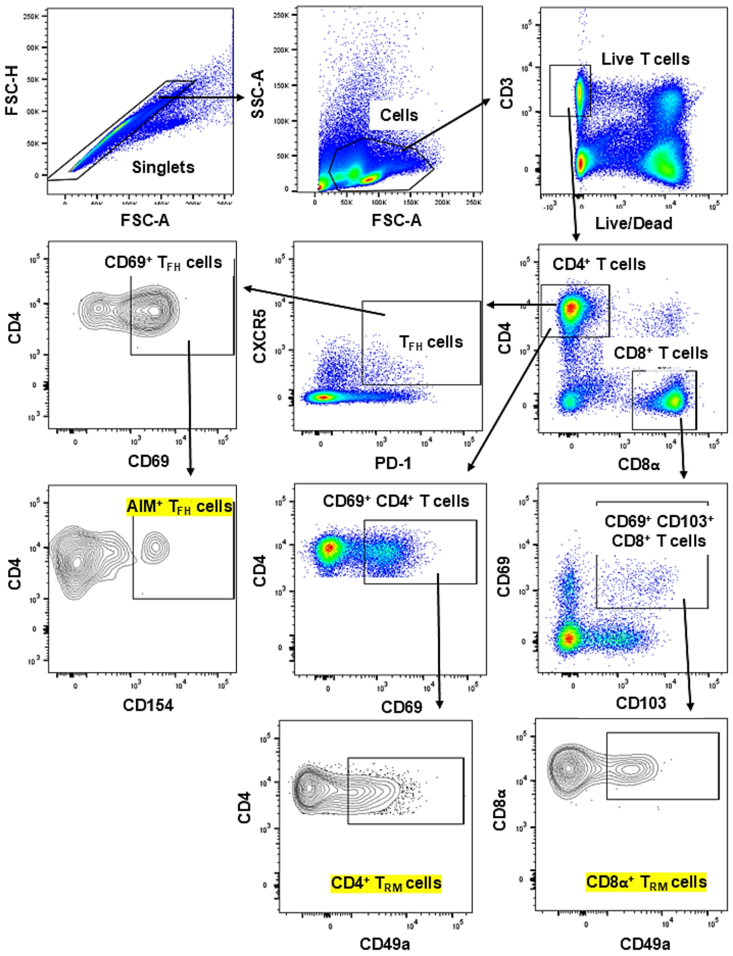

Figure S11. Gating strategy to identify AIM$^+$ T$_{FH}$ and T$_{RM}$ cells. Single cells were first identified via FSC-H/FSC-A and SSC-A/FSC-A. Dead cells were excluded with eFluor780 Fixable Viability Dye, and CD4$^+$ and CD8$^+$ T cells were identified from the total live T cell (Live/Dead$^-$ CD3$^+$) population. AIM$^+$ T$_{FH}$ cells were examined by first identifying total T$_{FH}$ cells (CXCR5$^+$ PD-1$^+$) from the CD4$^+$ T cell population, followed by gating on those that are double positive for both CD69 and CD154 AIM. T$_{RM}$ cells in lung and NALT tissues were identified from total CD4$^+$ and CD8$^+$ T cells by gating into CD69$^+$ and CD69$^+$ CD103$^+$ populations respectively, followed by further gating into cells that were also CD49a$^+$ (CD4$^+$: CD69$^+$ CD49a$^+$, CD8$^+$: CD69$^+$ CD103$^+$ CD49a$^+$).

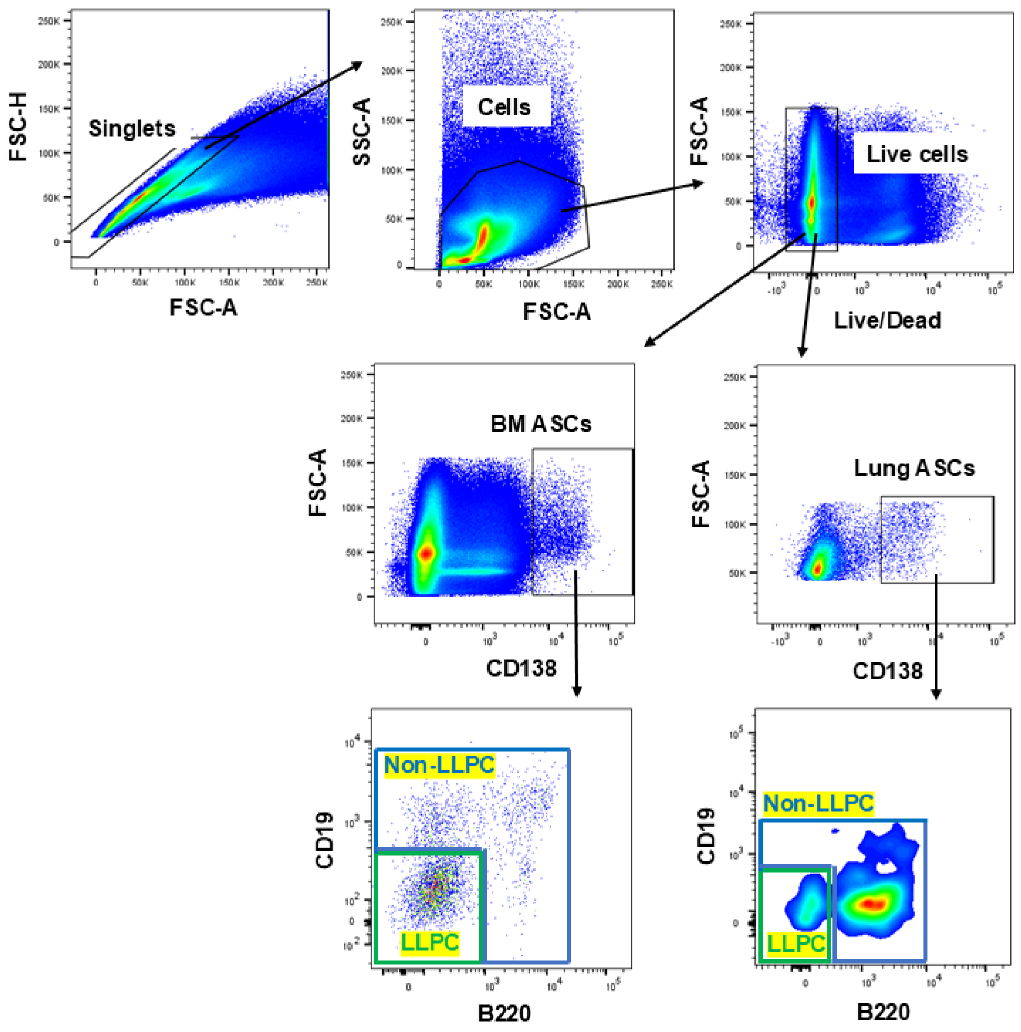

Figure S12. Gating strategy for sorting BM and lung LLPC and non-LLPC ASC subsets. Single cells were first identified via FSC-H/FSC-A and SSC-A/FSC-A. Dead cells were excluded with eFluor780 Fixable Viability Dye, and BM and lung ASC populations were identified as live cells that highly express CD138 (Live/Dead^{-} CD138^{hi}). Boolean gating was subsequently applied to identify LLPC and non-LLPC ASC subsets based on their expression of B220 and CD19; LLPC = CD138^{hi} B220^{lo} CD19^{lo} (green), non-LLPC = inverse of LLPC gating (blue).

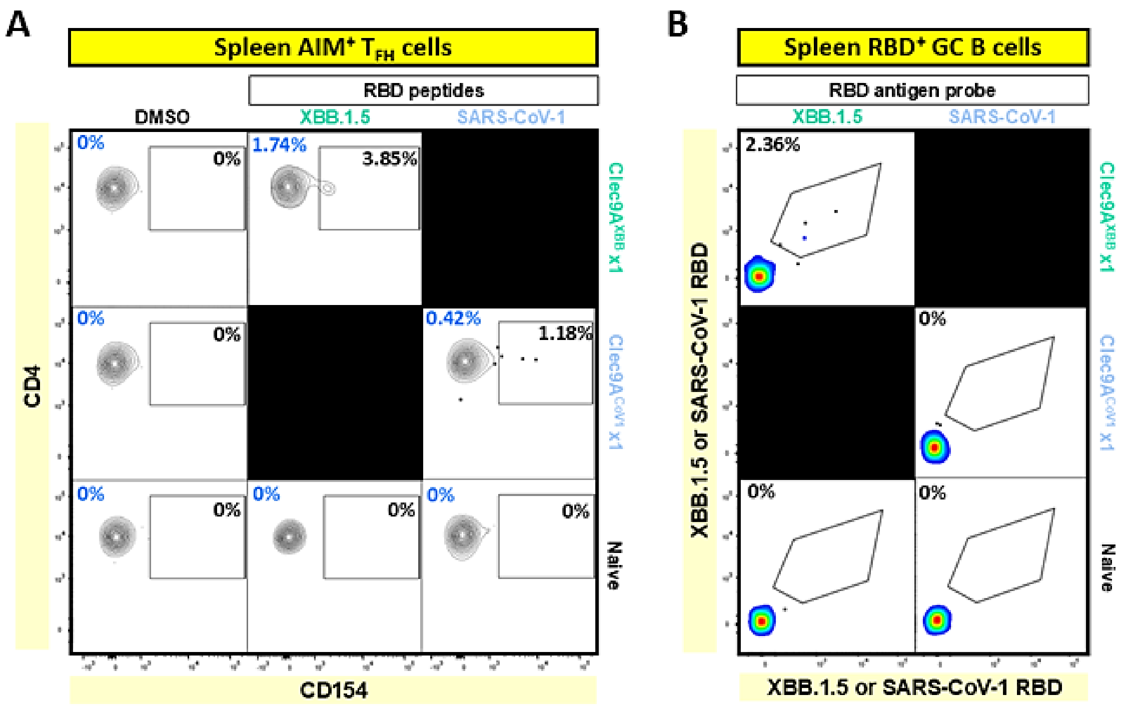

Figure S13. Representative flow cytometric analysis results of AIM$^+$ T$_{FH}$ and RBD$^+$ GC B cells following single dose Clec9A^{XBB} or Clec9A^{CoV1} immunization. **(A)** Representative plots of spleen AIM$^+$ T$_{FH}$ cells at six months post-boost. Values indicated in black and blue represent percentage AIM$^+$ T$_{FH}$ cells out of CD69$^+$ T$_{FH}$ cells and total T$_{FH}$ cells respectively. **(B)** Representative plots of RBD-specific spleen GC B cells at six months post-boost.

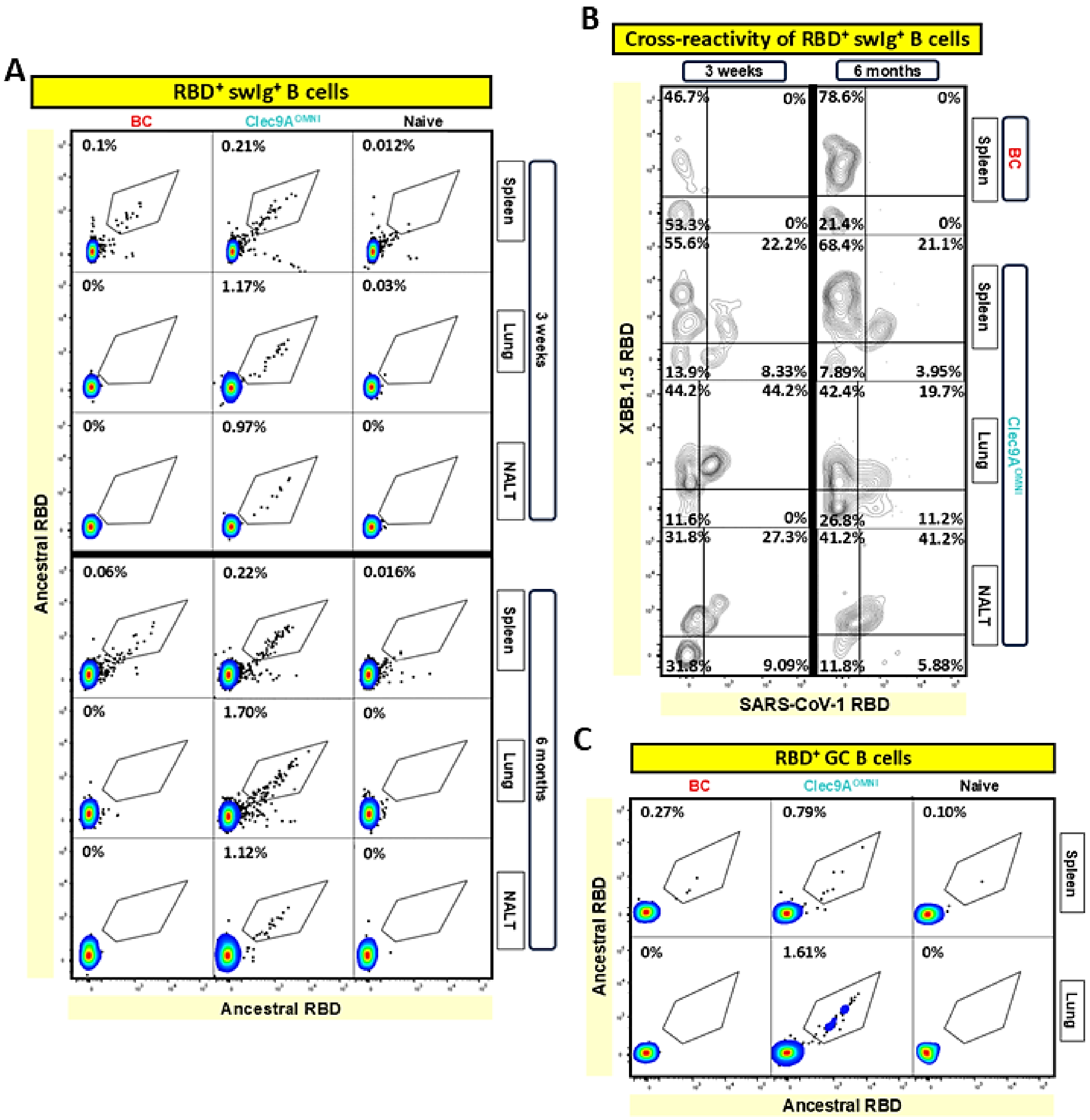

Figure S14. Representative flow cytometric analysis results of antigen-specific B cell subsets following BC mRNA vaccine and Clec9A^{OMNI} booster immunization. **(A, B)** Representative plots of ancestral SARS-CoV-2 RBD-specific spleen, lung and NALT **(A)** swIg^{+} B cells and their **(B)** cross-reactivity to XBB.1.5 and SARS-CoV-1 RBD, at three weeks and six months post-boost. **(C)** Representative plots of RBD-specific spleen and lung GC B cells at six months post-boost.

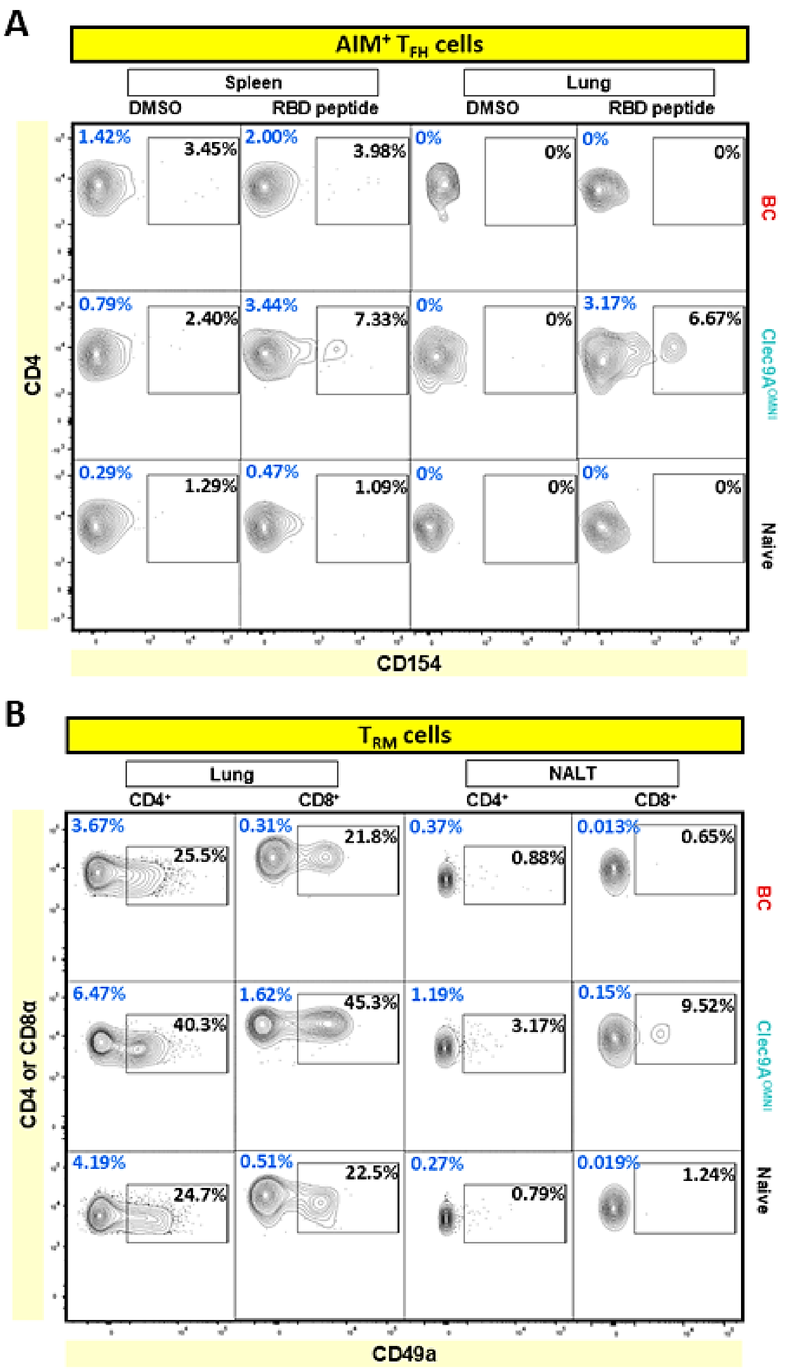

Figure S15. Representative flow cytometric analysis results of AIM$^+$ T$_{FH}$ and T$_{RM}$ cells following BC mRNA vaccine and Clec9A^{OMNI} booster immunization. **(A)** Representative plots of spleen and lung AIM$^+$ T$_{FH}$ cells at six months post-boost. Values indicated in black and blue represent percentage AIM$^+$ T$_{FH}$ cells out of CD69$^+$ T$_{FH}$ cells and total T$_{FH}$ cells respectively. **(B)** Representative plots of lung and NALT

$CD4^+$ and $CD8^+$ T_{RM} cells at one-month post-boost. Values indicated in black and blue represent percentage T_{RM} cells out of $CD69^+$ $CD4^+$ or $CD69^+$ $CD103^+$ $CD8^+$ T cells, and total $CD4^+$ or $CD8^+$ T cells respectively.

Table S1. Sarbecovirus RBD antigens used in the Multiplex sVNT.

Clade	Sarbecovirus	Variant/Strain	Source
1B	Pre-Omicron SARS-CoV-2	Ancestral	Custom made by Genscript
		Alpha	
		Beta	
		Delta	ACROBiosystems, SPD-C82Ed
		Delta Plus	Produced in-house in HEK293T cells as described previously (2)
		Lambda	
		Mu	
	Omicron SARS-CoV-2	BA.1	ACROBiosystems, SPD-C522K
		BA.2	ACROBiosystems, SPD-C82Eq
		BA.4/5	ACROBiosystems, SPD-C82Ew
		XBB.1.5	Produced in-house in HEK293T cells as described previously (2)
		EG.5	
		JN.1	
	Bat	RaTG13	Custom made by Genscript
		BANAL-52	Produced in-house in HEK293T cells as described previously (2)
		B21065	
	Pangolin	Gx-P5L	Custom made by Genscript
		GD-1	Produced in-house in HEK293T cells as described previously (2)
1A	Bat	Rs2018B	Produced in-house in HEK293T cells as described previously (2)
		RsSHC014	
		WIV-1	
	SARS-CoV-1	SARS-CoV-1	Custom made by Genscript

Table S2. Antibodies used for flow cytometry staining of antigen-specific B cell subsets, AIM^{+} T_{FH} cells, T_{RM} cells, and sorting of LLPC and non-LLPC ASC subsets.

Marker	Fluorophore	Manufacturer & catalogue #	Dilution/Concentration
**RBD^{+} swIg^{+} B cells**			
B220	Alexa Fluor 488	BD Biosciences, 557669	1:200
IgM	BUV395	BD Biosciences, 564025	
IgD		BD Biosciences, 564274	
Decoy (SAv)	BV605	BD Biosciences, 563260	1 µg/mL
Ancestral RBD	BV421	[redacted]	
	PE		
XBB.1.5 RBD	BV711		
SARS-CoV-1 RBD	APC		
**RBD^{+} GC B cells**			
B220	BUV395	BD Biosciences, 563793	1:200
IgD	BV711	BD Biosciences, 564275	
GL-7	Alexa Fluor 647	BD Biosciences, 561529	
CD95	BV605	Biolegend, 152612	
Decoy (SAv)	BB515	BD Biosciences, 564453	1 µg/mL
Ancestral RBD	BV421	[redacted]	
	PE		
**AIM^{+} T_{FH} cells**			
CD3	FITC	BD Biosciences, 555274	1:200
CD4	BUV395	BD Biosciences, 563790	
CXCR5	APC	BD Biosciences, 560615	
PD-1	BV605	BD Biosciences, 563059	
CD69	BV421	BD Biosciences, 562920	
CD154	PE	BD Biosciences, 553658	1:100, added in culture
**T_{RM} cells**			
CD3	FITC	BD Biosciences, 555274	1:200
CD4	BUV395	BD Biosciences, 563790	
CD8α	BV711	BD Biosciences, 563046	
CD69	BV421	BD Biosciences, 562920	
CD103	APC	BD Biosciences, 562772	
CD49a	PE	BD Biosciences, 562115	
**LLPC and non-LLPC ASC subset sorting**			
CD138	BV605	BD Biosciences, 563147	1:200
B220	Alexa Fluor 488	BD Biosciences, 557669	
CD19	BUV395	BD Biosciences, 563557	
